# Maternal Angiogenic Profiles in Preeclampsia-Associated Versus Isolated Fetal Growth Restriction or Small-for-Gestational-Age Pregnancies: A Systematic Review and Meta-Analysis

**DOI:** 10.3390/ijms27219829

**Published:** 2026-11-02

**Authors:** Min-A Kim, Eunju Lee, Hye Sun Lee, Ah-Young Choi

**Affiliations:** 1 Department of Obstetrics and Gynecology, Gangnam Severance Hospital, Yonsei University College of Medicine, Seoul 06273, Republic of Korea; cay423@yuhs.ac; 2 Biostatistics Collaboration Unit, Yonsei University College of Medicine, Seoul 06273, Republic of Korea; dmswn1578@yuhs.ac (E.L.); hslee1@yuhs.ac (H.S.L.)

**Keywords:** preeclampsia, fetal growth restriction, small for gestational age, placental growth factor, soluble fms-like tyrosine kinase-1, sFlt-1/PlGF ratio, angiogenic biomarkers

## Abstract

Maternal angiogenic imbalance is a hallmark of preeclampsia (PE), but whether it differs between PE-associated and isolated fetal growth restriction (FGR)/small-for-gestational-age (SGA) pregnancies is unclear, particularly before clinical recognition. We searched PubMed, Embase, and CENTRAL for observational studies comparing maternal soluble fms-like tyrosine kinase-1 (sFlt-1), placental growth factor (PlGF), or their ratio between these phenotypes. Analyses were stratified by sampling context and pooled as Hedges’ g using random-effects models. Twenty-three studies (630 PE-associated, 1476 isolated FGR/SGA pregnancies) were included. After clinical recognition, PE-associated FGR/SGA showed a higher sFlt-1/PlGF ratio (g = 1.04, 95% CI 0.51 to 1.57), higher sFlt-1 (1.05, 0.73 to 1.38), and lower PlGF (−0.60, −0.82 to −0.38) than isolated FGR/SGA; differences in the same directions were present before clinical recognition (ratio 0.86, 0.38 to 1.33; sFlt-1 0.38, 0.12 to 0.64; PlGF −0.46, −0.77 to −0.16). The sFlt-1 difference was larger after clinical recognition in the test for subgroup differences (*p* = 0.002) but not in meta-regression (*p* = 0.064), and this between-context contrast is therefore reported as exploratory; the PlGF difference was similar in both contexts. These group-level differences do not establish individual-level prediction, discrimination or threshold performance. PE-associated and isolated FGR/SGA should therefore be distinguished in biomarker research, and angiogenic thresholds derived in suspected PE require validation before application to normotensive FGR/SGA.

## 1. Introduction

Preeclampsia (PE) affects approximately 2–8% of pregnancies, whereas fetal growth restriction (FGR) is estimated to complicate 5–10%, although reported frequencies vary according to the population and diagnostic criteria [1,2]. Both conditions are major causes of maternal and perinatal morbidity and mortality and frequently coexist in early-onset placental disease. FGR may occur as part of the maternal hypertensive syndrome or in an otherwise normotensive pregnancy [3]. Although FGR and small for gestational age (SGA) are not equivalent concepts—SGA describes size and includes constitutionally small infants, whereas FGR denotes failure to achieve biological growth potential [4]—both have been used to define impaired fetal growth in the angiogenic-biomarker literature.

The distinction between PE-associated and isolated FGR/SGA has direct clinical relevance. Once maternal hypertensive disease is present, management may differ because delivery can be indicated on maternal grounds rather than on fetal criteria alone. Angiogenic tests currently used in clinical practice were developed and validated primarily in women with suspected PE, whereas their role in normotensive FGR/SGA remains uncertain [5,6]. Prediction and screening studies have also reported biomarker associations or predictive performance for composite placental outcomes that include both PE and impaired fetal growth, or for PE-related outcomes in which the presence or absence of FGR/SGA was not specified [7]. Such analyses cannot determine whether the observed biomarker signal reflects PE, impaired fetal growth, or their coexistence. Consequently, a signal driven predominantly by PE-associated cases may not generalise to normotensive FGR/SGA.

Placental forms of PE and FGR share impaired trophoblast invasion and inadequate spiral-artery remodelling, leading to placental malperfusion, oxidative stress, and angiogenic imbalance; in PE, these placental disturbances are accompanied by maternal endothelial dysfunction [8,9]. The best-characterised circulating markers are soluble fms-like tyrosine kinase-1 (sFlt-1), an antiangiogenic soluble receptor, and placental growth factor (PlGF), a placenta-derived proangiogenic factor. Increased sFlt-1, reduced PlGF, and an increased sFlt-1/PlGF ratio are established features of PE, with either ratio-based or PlGF-only testing used according to the assay platform and clinical setting [6,10]. Because sFlt-1 released into the maternal circulation antagonises PlGF and contributes to endothelial dysfunction and hypertension [8,9], the circulating imbalance is expected to be more pronounced when placental dysfunction is accompanied by the maternal syndrome of PE. In a normotensive pregnancy, placental dysfunction may lower PlGF without the same degree of sFlt-1 excess [11]. Both phenotypes may therefore differ from uncomplicated pregnancy while differing from each other in the magnitude of the imbalance and in the relative contribution of the two markers. However, it remains uncertain whether PE-associated and isolated FGR/SGA represent quantitatively or qualitatively distinct maternal angiogenic phenotypes.

Direct comparison of these two impaired-growth phenotypes may help distinguish the additional angiogenic disturbance associated with maternal PE. Most biomarker studies instead compare affected with unaffected pregnancies, a design in which impaired fetal growth and maternal PE are not separated. Differences observed between the two impaired-growth phenotypes may more specifically reflect angiogenic alterations associated with maternal PE, although residual confounding cannot be excluded. The same question arises in practice when a small fetus is identified in a normotensive woman and angiogenic testing is considered, since the available thresholds were derived in women with suspected PE. However, previous studies have reported variable differences between these phenotypes, applied heterogeneous definitions of FGR and SGA, and obtained samples at different stages of disease [12,13]. Reported findings range from similarity to large differences: in one study of samples obtained close to delivery, the authors concluded that the angiogenic imbalance reflects placental disease regardless of its maternal clinical manifestation [13], whereas others reported an sFlt-1/PlGF ratio several-fold higher when PE accompanied impaired growth [14,15]. An earlier study found sFlt-1 to be increased in PE but not in normotensive pregnancies with SGA neonates [16]. These reports also differ in the growth-outcome definition applied and in when samples were obtained relative to clinical recognition. These differences in study design make it difficult to determine whether the inconsistent findings reflect genuine differences between phenotypes or methodological heterogeneity. To our knowledge, this evidence has not previously been quantitatively synthesised with stratification by clinical sampling context.

We hypothesised that PE-associated FGR/SGA is characterised by a more antiangiogenic maternal profile than isolated FGR/SGA, with higher sFlt-1, lower PlGF and a higher sFlt-1/PlGF ratio, and that this difference is present both before and after clinical recognition. We therefore conducted a systematic review and meta-analysis comparing maternal sFlt-1, PlGF, and the sFlt-1/PlGF ratio between PE-associated and isolated FGR/SGA. Separate analyses were performed for samples obtained before and after clinical recognition to determine whether the two phenotypes exhibit distinct maternal angiogenic profiles in each sampling context.

## 2. Results

### 2.1. Study Selection and Characteristics

**Figure 1:**
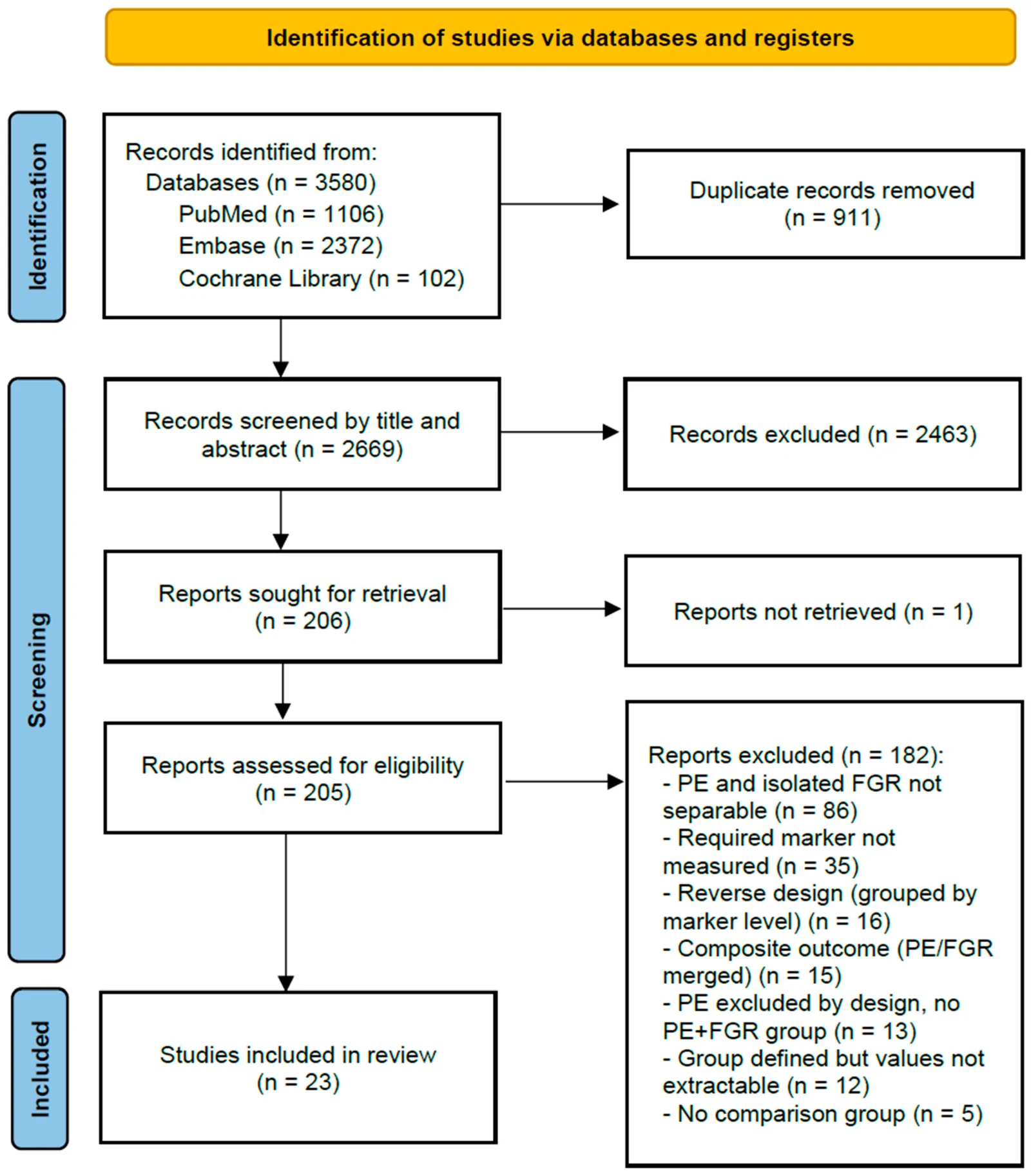
PRISMA 2020 flow diagram of study identification, screening, eligibility assessment, and inclusion. PE, preeclampsia; FGR, fetal growth restriction.

The study-selection process is presented in the PRISMA 2020 flow diagram (Figure 1). Twenty-three studies [13,14,15,16,17,18,19,20,21,22,23,24,25,26,27,28,29,30,31,32,33,34,35] published between 2005 and 2026 were included in the meta-analysis (Table 1). Nine studies evaluated biomarkers before clinical recognition, and fourteen evaluated biomarkers after clinical recognition; each study contributed to only one sampling context. Overall, 630 pregnancies with PE-associated FGR/SGA and 1476 with isolated FGR/SGA were included (sampling before clinical recognition, 155 and 597; sampling after clinical recognition, 475 and 879).

Thirteen studies defined FGR using antenatal fetal biometry and/or Doppler criteria, whereas ten used birthweight-based SGA criteria. Markers were measured by automated immunoassay in thirteen studies and by research ELISA in ten, and two studies reported multiples of the median rather than absolute concentrations.

The preeclampsia definition required proteinuria in 15 studies and admitted maternal organ dysfunction without proteinuria in 6; 2 did not report diagnostic criteria in sufficient detail to classify (Supplementary Table S1).

Of the 14 studies sampling after clinical recognition, both PE and FGR/SGA had been recognised at the time of sampling in 8, FGR/SGA had been recognised without confirmation of PE in 2, PE had been recognised without confirmation of FGR/SGA in 3, and the statuses were mixed within the study population in 1; no study was indeterminate for both conditions (Supplementary Table S1).

Of the 23 included studies, eight reported group-specific gestational ages at blood sampling, nine provided only a common sampling window, two provided partial information, and four did not provide sufficient information. Among studies reporting group-specific values, the difference between comparison groups (PE-associated minus isolated FGR/SGA) ranged from −4.0 to +1.2 weeks (Supplementary Table S1). Of the 50 effect estimates, 35 were derived from medians and interquartile ranges, 13 were taken from directly reported means and standard deviations, and 2 were derived from a reported standard error.

### 2.2. sFlt-1/PlGF Ratio

**Figure 2:**
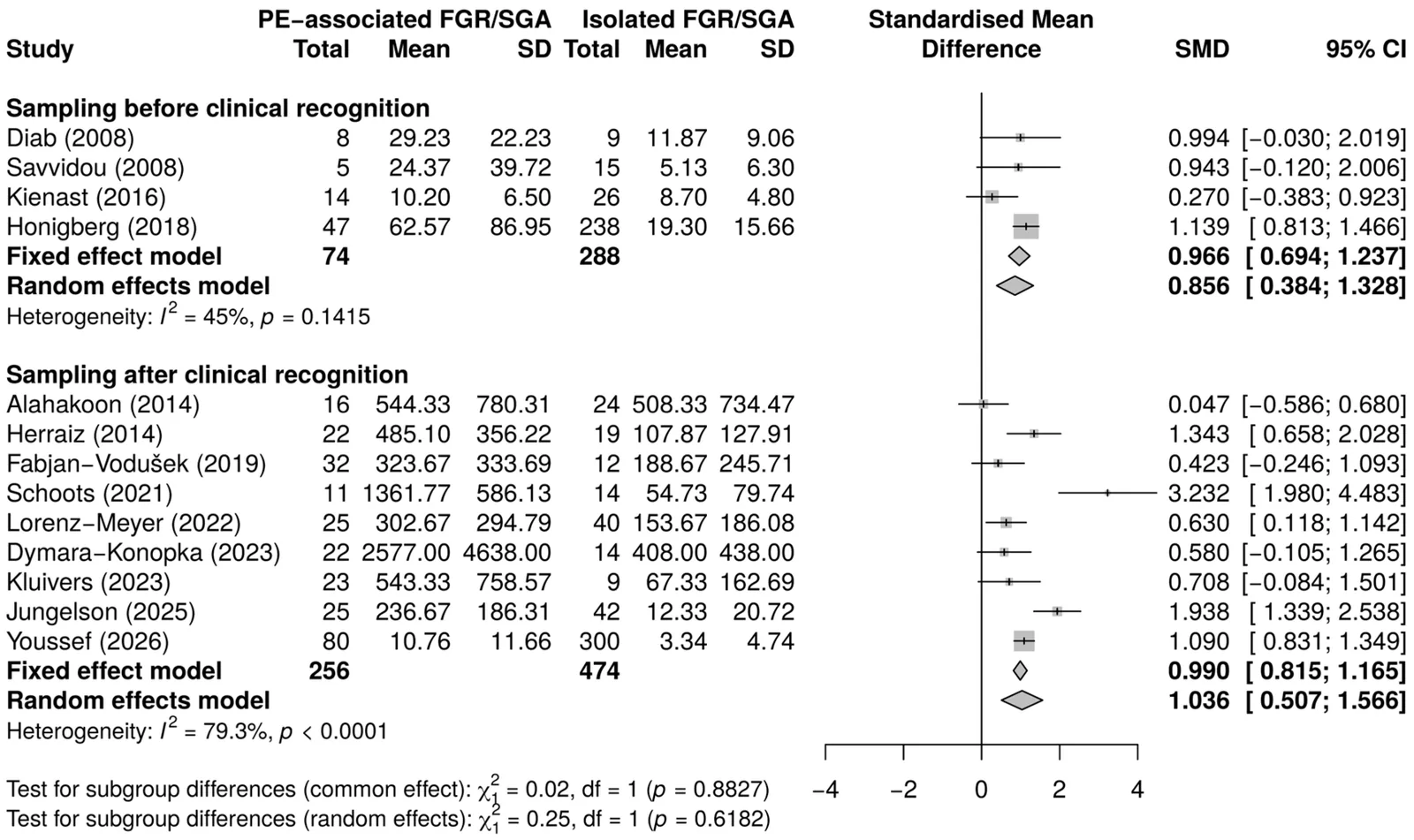
Forest plot of sFlt-1/PlGF ratio in PE-associated versus isolated FGR/SGA, by clinical sampling context. PE, preeclampsia; FGR, fetal growth restriction; SGA, small for gestational age. Standardised mean differences (SMDs; Hedges’ g) compare maternal sFlt-1/PlGF ratios between PE-associated and isolated FGR/SGA, with positive values indicating higher ratios in PE-associated FGR/SGA. Squares represent study-specific effect estimates, with their size reflecting study weight; horizontal lines indicate 95% confidence intervals (CIs). Diamonds represent pooled estimates. Random-effects estimates were used as the principal results, with fixed-effect estimates shown for comparison. Data are from the included studies [13,14,15,19,20,23,25,26,27,29,30,33,35].

Before clinical recognition, four studies including 74 pregnancies that subsequently developed PE-associated FGR/SGA and 288 that subsequently developed isolated FGR/SGA contributed to the pooled analysis. The maternal sFlt-1/PlGF ratio was higher in pregnancies that subsequently developed PE-associated FGR/SGA than in those that subsequently developed isolated FGR/SGA (Hedges’ g = 0.86, 95% CI 0.38 to 1.33; *p* < 0.001; Figure 2), with study-level estimates ranging from 0.27 to 1.14 and moderate heterogeneity that was not statistically significant (I^2^ = 45.0%, Cochran’s Q *p* = 0.141).

After clinical recognition, nine studies including 256 pregnancies with PE-associated FGR/SGA and 474 with isolated FGR/SGA contributed to the pooled analysis. The ratio was higher in pregnancies with PE-associated FGR/SGA than in those with isolated FGR/SGA (Hedges’ g = 1.04, 95% CI 0.51 to 1.57; *p* < 0.001). The direction of effect was positive in every contributing study, with study-level estimates ranging from 0.05 to 3.23. Between-study heterogeneity was considerable (I^2^ = 79.3%, Cochran’s Q *p* < 0.001), indicating substantial variation in the magnitude of the difference across studies. With heterogeneity of this degree, the pooled value describes the average difference across a varied evidence base rather than a uniform effect, and the magnitude in any single setting is not estimated by it.

### 2.3. sFlt-1

**Figure 3:**
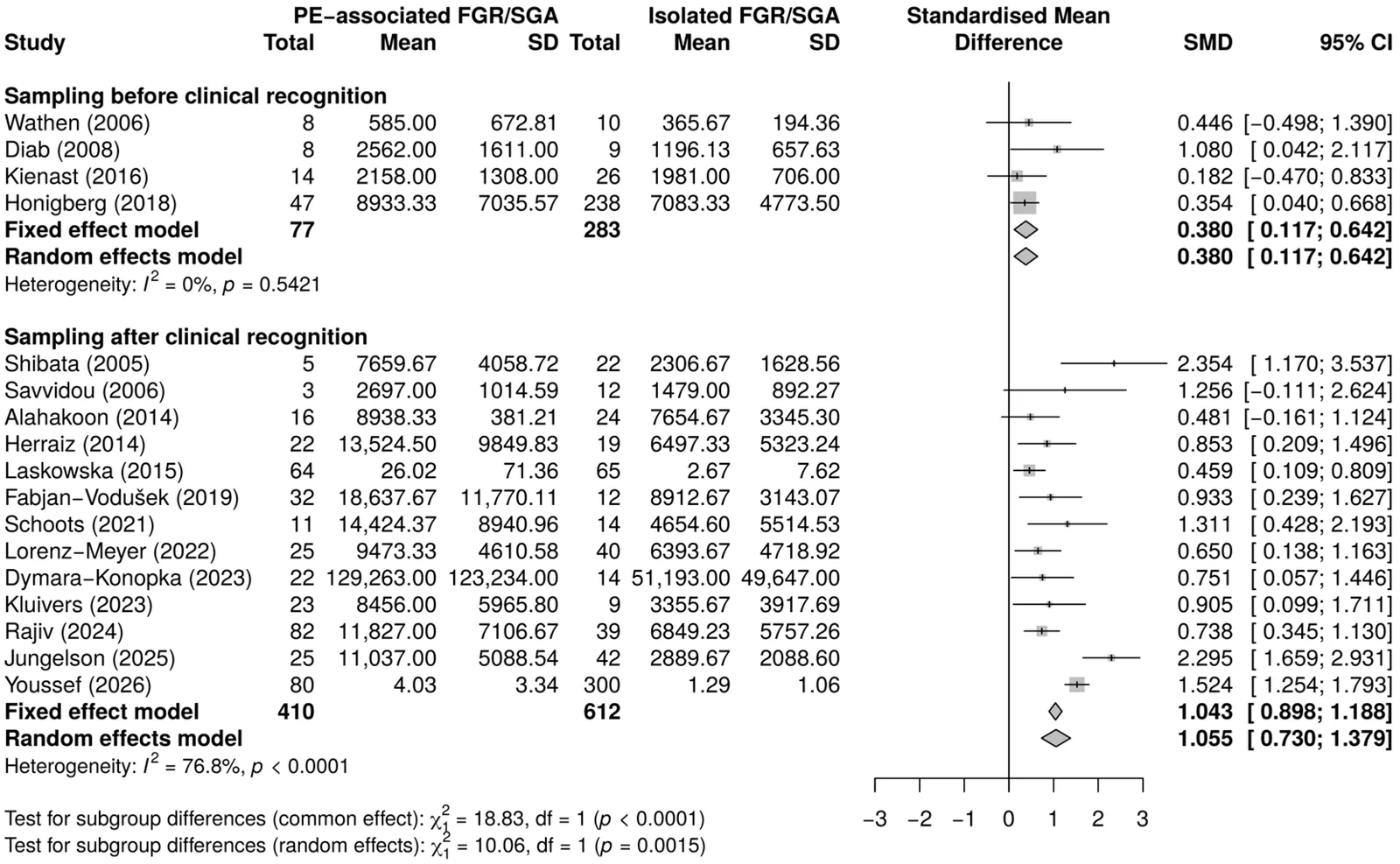
Forest plot of sFlt-1 in PE-associated versus isolated FGR/SGA, by clinical sampling context. PE, preeclampsia; FGR, fetal growth restriction; SGA, small for gestational age. Standardised mean differences (SMDs; Hedges’ g) compare maternal sFlt-1 levels between PE-associated and isolated FGR/SGA, with positive values indicating higher levels in PE-associated FGR/SGA. Squares represent study-specific effect estimates, with their size reflecting study weight; horizontal lines indicate 95% confidence intervals (CIs). Diamonds represent pooled estimates. Random-effects estimates were used as the principal results, with fixed-effect estimates shown for comparison. Values reported by Youssef (2026) [35] are expressed as multiples of the median (MoM) rather than absolute concentrations. Data are from the included studies [13,14,15,16,17,18,19,23,24,25,26,27,29,30,31,33,35].

Before clinical recognition, four studies including 77 pregnancies that subsequently developed PE-associated FGR/SGA and 283 that subsequently developed isolated FGR/SGA contributed to the pooled analysis. Maternal sFlt-1 concentrations were higher in pregnancies that subsequently developed PE-associated FGR/SGA than in those that subsequently developed isolated FGR/SGA (g = 0.38, 95% CI 0.12 to 0.64; *p* = 0.005; Figure 3), with study-level estimates ranging from 0.18 to 1.08 and no detectable statistical heterogeneity (I^2^ = 0%, Cochran’s Q *p* = 0.542).

After clinical recognition, thirteen studies including 410 pregnancies with PE-associated FGR/SGA and 612 with isolated FGR/SGA contributed to the pooled analysis. sFlt-1 was higher in pregnancies with PE-associated FGR/SGA than in those with isolated FGR/SGA (g = 1.05, 95% CI 0.73 to 1.38; *p* < 0.001), with considerable heterogeneity (I^2^ = 76.8%, Cochran’s Q *p* < 0.001) and study-level estimates ranging from 0.46 to 2.35. As for the ratio, the pooled value describes the average difference across studies that differ considerably in magnitude and should not be read as the difference to be expected in a particular population.

### 2.4. PlGF

**Figure 4:**
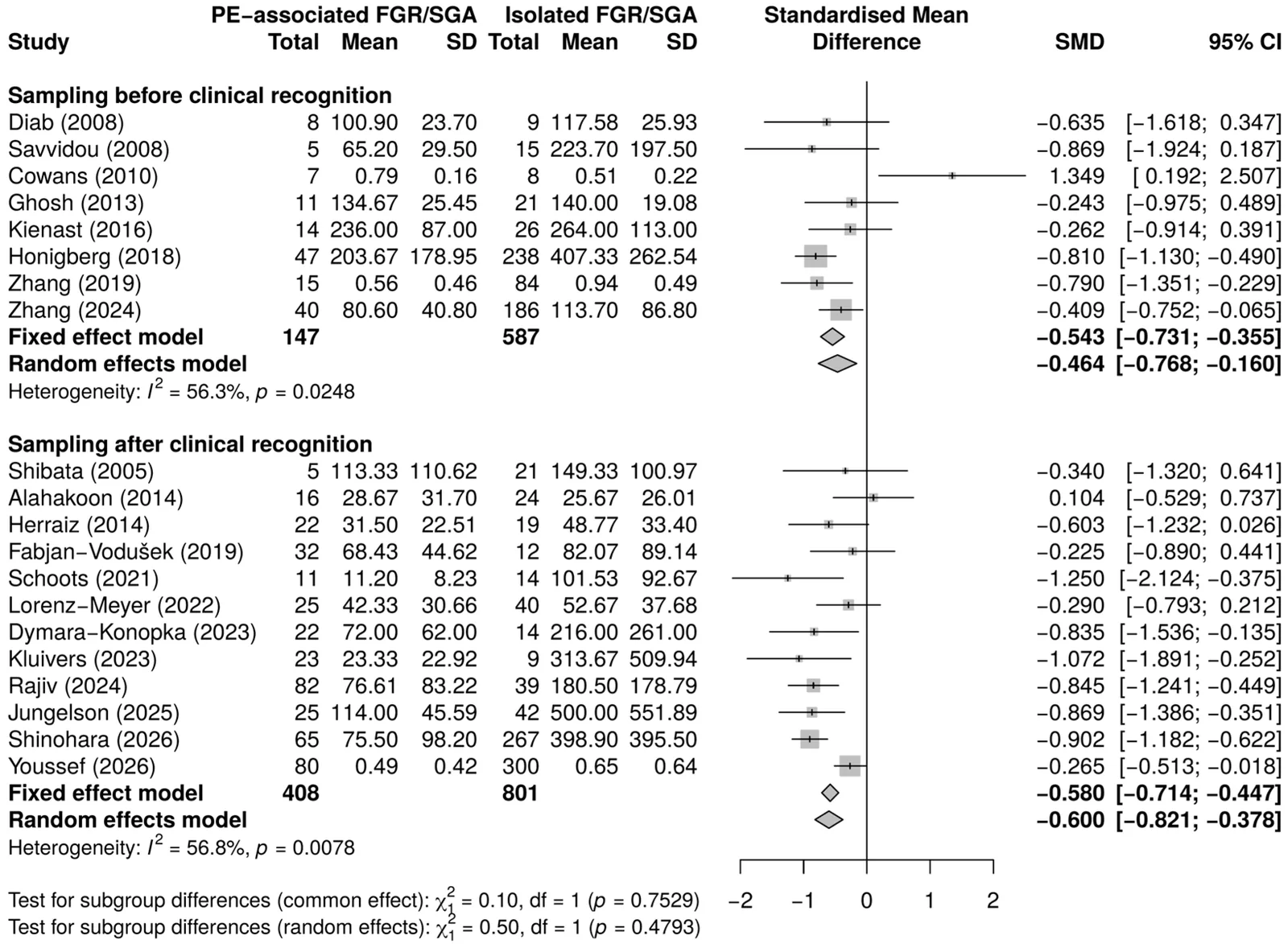
Forest plot of PlGF in PE-associated versus isolated FGR/SGA, by clinical sampling context. PE, preeclampsia; FGR, fetal growth restriction; SGA, small for gestational age. Standardised mean differences (SMDs; Hedges’ g) compare maternal PlGF levels between PE-associated and isolated FGR/SGA, with positive values indicating higher levels in PE-associated FGR/SGA. Squares represent study-specific effect estimates, with their size reflecting study weight; horizontal lines indicate 95% confidence intervals (CIs). Diamonds represent pooled estimates. Random-effects estimates were used as the principal results, with fixed-effect estimates shown for comparison. Values reported by Cowans (2010) [21] and Youssef (2026) [35] are expressed as multiples of the median (MoM) rather than absolute concentrations. Data are from the included studies [13,14,15,16,19,20,21,22,23,25,26,27,28,29,30,31,32,33,34,35].

Before clinical recognition, eight studies, including 147 pregnancies that subsequently developed PE-associated FGR/SGA and 587 that subsequently developed isolated FGR/SGA, contributed to the pooled analysis. Maternal PlGF concentrations were lower in pregnancies that subsequently developed PE-associated FGR/SGA than in those that subsequently developed isolated FGR/SGA (g = −0.46, 95% CI −0.77 to −0.16; *p* = 0.003; Figure 4), with moderate heterogeneity (I^2^ = 56.3%, Cochran’s Q *p* = 0.025) and study-level estimates ranging from −0.87 to 1.35.

After clinical recognition, twelve studies including 408 pregnancies with PE-associated FGR/SGA and 801 with isolated FGR/SGA contributed to the pooled analysis. PlGF was lower in pregnancies with PE-associated FGR/SGA than in those with isolated FGR/SGA (g = −0.60, 95% CI −0.82 to −0.38; *p* < 0.001), with moderate heterogeneity (I^2^ = 56.8%, Cochran’s Q *p* = 0.008) and study-level estimates ranging from −1.25 to 0.10.

### 2.5. Differences Between Sampling Contexts

Group-level differences between PE-associated and isolated FGR/SGA were observed for all three markers in both sampling contexts. The PlGF difference was similar in magnitude before and after clinical recognition (Hedges’ g = −0.46 and −0.60, respectively), whereas the sFlt-1 difference was substantially larger after clinical recognition (0.38 and 1.05, respectively). In both contexts, the higher sFlt-1/PlGF ratio in PE-associated FGR/SGA was accompanied by both higher sFlt-1 and lower PlGF.

The test for subgroup differences indicated that the sFlt-1 effect differed between the two sampling contexts ($\chi_{1}^{2}$ = 10.06, *p* = 0.002), whereas no significant between-context difference was found for PlGF ($\chi_{1}^{2}$ = 0.50, *p* = 0.479) or the sFlt-1/PlGF ratio ($\chi_{1}^{2}$ = 0.25, *p* = 0.618). Meta-regression with sampling context as a study-level moderator yielded a positive but non-significant coefficient for sFlt-1 (B = 0.60, 95% CI −0.03 to 1.23; *p* = 0.064) and showed no association for PlGF (B = −0.13, 95% CI −0.50 to 0.24; *p* = 0.488) or the ratio (B = 0.19, 95% CI −0.66 to 1.03; *p* = 0.666). These between-context comparisons were considered exploratory (Table 2).

### 2.6. Exploratory Moderator Analyses

Of the 30 possible marker–moderator combinations across the two sampling contexts, 25 were estimable and analysed using univariable meta-regression without adjustment for multiplicity (Supplementary Table S3). In the analyses before clinical recognition, gestational age at sampling was positively associated with the effect estimate for the sFlt-1/PlGF ratio (B = 0.17 per week, 95% CI 0.02 to 0.32; *p* = 0.028). No statistically significant associations were observed for the other estimable moderators (all *p* ≥ 0.172). Maternal age could not be evaluated for sFlt-1 or the ratio before clinical recognition because data were available from only two studies.

In the analyses after clinical recognition, FGR/SGA definition was associated with the sFlt-1 effect estimate (B = 0.95 for birthweight-defined SGA relative to antenatally defined FGR, 95% CI 0.25 to 1.66; *p* = 0.008); the corresponding test for subgroup differences gave *p* = 0.058 (Supplementary Table S5), reflecting the difference in between-study variance assumptions described in Section 4.6. Assay class was associated with the PlGF effect estimate (B = 0.47 for research ELISA relative to automated immunoassay, 95% CI 0.13 to 0.80; *p* = 0.006). No statistically significant associations were identified for the other estimable moderators (all *p* ≥ 0.116). Clinical status at the time of sampling was not evaluated because it did not vary within the analyses after clinical recognition. All moderator findings were considered exploratory and hypothesis-generating: the models contained small numbers of contributing studies, no adjustment was made for multiplicity across the 25 estimable models, and none of the associations was used to support a mechanistic interpretation.

### 2.7. Risk of Bias and Small-Study Effects

The cohort version of the Newcastle–Ottawa Scale was applied to 13 studies and the case–control version to 10. Scores ranged from 4 to 9 of a maximum of 9 stars (median 7); item-level assessments and the version applied to each study are provided in Supplementary Table S4. Ratings were most limited in the comparability domain: 2 studies received two stars, 11 received one, and 10 received none, indicating that control for gestational age at sampling and other potential confounders was generally incomplete. Selection scores ranged from 2 to 4 stars (maximum 4) and outcome or exposure scores from 1 to 3 stars (maximum 3).

Egger’s regression tests were not statistically significant in any of the six pooled analyses, with *p*-values ranging from 0.235 to 0.912 (Supplementary Figure S1). These findings were interpreted cautiously because four of the six analyses included fewer than ten studies and therefore had limited power to detect funnel-plot asymmetry. Non-significant Egger tests were not considered evidence that small-study effects or publication bias were absent.

### 2.8. Sensitivity Analyses

Pooled estimates calculated separately by growth-outcome definition, with the corresponding tests for subgroup differences, are presented in Supplementary Table S5. After clinical recognition, all three markers differed in the same directions within both definition strata, and the estimates among antenatally defined FGR studies alone were 0.95 (95% CI 0.33 to 1.58) for the sFlt-1/PlGF ratio, 0.87 (0.60 to 1.15) for sFlt-1 and −0.55 (−0.81 to −0.30) for PlGF. No test for subgroup differences between definitions reached statistical significance. Before clinical recognition, the antenatally defined stratum contained one study for the ratio, and for sFlt-1 and two for PlGF, so only the PlGF comparison could be pooled, and no definition-specific inference was drawn in that context.

Among studies reporting group-specific sampling gestational ages, the pooled effects after clinical recognition were 1.14 (95% CI 0.43 to 1.85) for the sFlt-1/PlGF ratio, 1.03 (0.55 to 1.50) for sFlt-1, and −0.63 (−0.97 to −0.30) for PlGF. Restriction to studies with an absolute between-group difference of no more than 1.5 weeks yielded corresponding estimates of 1.16 (0.54 to 1.78), 1.17 (0.42 to 1.93), and −0.65 (−0.99 to −0.32), respectively. Meta-regression did not identify significant associations between sampling gestational-age differences and biomarker effect sizes (all *p* ≥ 0.38). Neither analysis could be performed before clinical recognition, where only one study reported group-specific sampling gestational age. In the analysis before clinical recognition restricted to studies with documented sampling gestational-age comparability or gestational-age-standardised measurements, the PlGF estimate was imprecise (g = 0.01, 95% CI −1.17 to 1.19; k = 3; I^2^ = 81.4%; Supplementary Table S6).

Omitting each study in turn left the three analyses after clinical recognition statistically significant throughout, as were the ratio and PlGF analyses before clinical recognition; sFlt-1 before clinical recognition ceased to be significant when Honigberg, 2018 [26] was omitted. Excluding studies with fewer than 10 PE-associated cases left the analyses after clinical recognition essentially unchanged (1.04, 0.98 and −0.61 for the ratio, sFlt-1 and PlGF) and reduced the ratio analysis before clinical recognition to two studies, in which it was no longer significant (0.75, 95% CI −0.09 to 1.60). Excluding case–control designs, and separately excluding studies scoring below the median of 7 of 9 Newcastle–Ottawa stars, left all six analyses significant and in the same directions; the latter restriction left two to four studies per analysis after clinical recognition (Supplementary Table S6).

Restriction to directly reported means and standard deviations left one to four studies per analysis; the direction of effect was preserved wherever an estimate could be calculated, but the estimates were imprecise and are reported descriptively (Supplementary Table S6).

All six pooled effects remained statistically significant under Hartung–Knapp inference, with wider intervals than the Wald-type intervals (*p* = 0.026 to 0.044 before and *p* ≤ 0.008 after clinical recognition). Hartung–Knapp intervals for all six analyses are reported in Supplementary Table S6. Ninety-five percent prediction intervals included the null in all six analyses (Table 2).

## 3. Discussion

### 3.1. Principal Findings

In this systematic review and meta-analysis of 23 studies comprising 630 pregnancies with PE-associated FGR/SGA and 1476 with isolated FGR/SGA, all three angiogenic markers differed between the two phenotypes in both sampling contexts. After clinical recognition, the maternal sFlt-1/PlGF ratio and sFlt-1 were higher, and PlGF was lower in PE-associated FGR/SGA than in isolated FGR/SGA. Before clinical recognition, pregnancies that subsequently developed PE-associated FGR/SGA likewise had a higher sFlt-1/PlGF ratio and higher sFlt-1, together with lower PlGF, than pregnancies that subsequently developed isolated FGR/SGA. The two phenotypes therefore showed differences in their maternal angiogenic profiles, and these differences were not confined to the period after the maternal syndrome became clinically apparent.

Four of the six analyses were based on fewer than ten studies, namely the three analyses before clinical recognition and the sFlt-1/PlGF ratio after clinical recognition; their pooled magnitudes should therefore be read as imprecise estimates rather than as established effect sizes.

The findings after clinical recognition did not depend on the inclusion of birthweight-defined studies: all three were reproduced within the antenatally defined FGR studies alone (Section 2.8). The sFlt-1 difference was larger among the birthweight-defined studies, but the test for subgroup differences between definitions was not statistically significant, so no firm conclusion is drawn about the influence of growth-outcome definition on effect magnitude.

The two component markers, PlGF and sFlt-1, showed different patterns across the two sampling contexts. The PlGF difference was of similar magnitude before and after clinical recognition (Hedges’ g = −0.46 and −0.60, respectively), whereas the sFlt-1 difference was substantially larger after clinical recognition (Hedges’ g = 0.38 and 1.05, respectively). Sampling context did not reach statistical significance as a study-level moderator in meta-regression (B = 0.60, 95% CI −0.03 to 1.23; *p* = 0.064), whereas the test for subgroup differences was significant ($\chi_{1}^{2}$ = 10.06, *p* = 0.002). The two procedures differ in their treatment of between-study variance: τ^2^ was estimated separately within each context in the subgroup test (0.000 before and 0.238 after clinical recognition) and as a single common value in meta-regression (0.192). With four studies before and thirteen studies after clinical recognition, τ^2^ is itself imprecisely estimated, so neither assumption can be preferred on the available evidence. The between-context comparison was therefore sensitive to the statistical approach and does not provide robust evidence of a difference in sFlt-1 effects between sampling contexts.

Taken together, these findings indicate that the pooled sFlt-1 difference between PE-associated and isolated FGR/SGA was numerically larger in studies sampling after clinical recognition than in those sampling before clinical recognition. However, the evidence for a difference between sampling contexts was not consistent across statistical approaches, and the estimates were derived from different studies and participants. Therefore, this pattern should not be interpreted as a longitudinal change within individual pregnancies.

### 3.2. Biological Interpretation of sFlt-1 and PlGF

The differences observed after clinical recognition were in the expected direction and were consistent with the known angiogenic changes in PE [36]; the corresponding differences before clinical recognition were the less anticipated findings.

The contrasting patterns of PlGF and sFlt-1 may provide insight into the angiogenic differences between PE-associated and isolated FGR/SGA. PlGF is predominantly produced by the placenta and is involved in placental angiogenesis, whereas sFlt-1 acts as an antiangiogenic factor by binding circulating PlGF and VEGF [9,37]. In the present analysis, lower PlGF in PE-associated FGR/SGA was present before clinical recognition, and the magnitude of this difference remained broadly similar after clinical recognition. By contrast, the between-group difference in sFlt-1 was relatively modest before clinical recognition but substantially larger after clinical recognition. This pattern is compatible with the possibility that differences in the proangiogenic component of the angiogenic balance may be detectable before clinical recognition, whereas a larger antiangiogenic difference is observed in studies sampling after clinical recognition. Because the clinical status of participants at the time of sampling was not uniformly reported, and because some samples were obtained after the recognition of fetal growth restriction without confirmation that preeclampsia had already been diagnosed, the larger sFlt-1 difference cannot be attributed specifically to a clinically established maternal PE phenotype.

This biological interpretation remains inferential. In particular, the findings do not demonstrate temporal progression from PlGF reduction to sFlt-1 elevation. Studies sampling after clinical recognition may also have captured more severe placental disease overall, with both the placental lesion and the maternal response being more advanced.

### 3.3. Findings Before Clinical Recognition

Angiogenic markers have been extensively studied in PE, and individual studies have also examined these markers in normotensive FGR/SGA [11]. To our knowledge, no previous meta-analysis has quantitatively compared PE associated with isolated FGR/SGA while distinguishing between measurements obtained before clinical recognition and those obtained after it. The differences observed before clinical recognition are particularly relevant because biomarker measurement preceded the diagnosis of either PE or FGR/SGA. In that context, all three markers differed in the same direction as after clinical recognition. This finding suggests that the subsequent coexistence of PE and impaired fetal growth is preceded by a different maternal angiogenic milieu from that preceding isolated FGR/SGA, rather than the observed difference arising only after clinically apparent PE.

These findings should not, however, be interpreted as evidence of individual-level prediction or discrimination. The meta-analysis compared group-level biomarker distributions and did not evaluate a common threshold, sensitivity, specificity, or incremental predictive performance over established clinical risk factors. Moreover, the evidence base before clinical recognition was relatively small, particularly for sFlt-1 and the sFlt-1/PlGF ratio, for which only four studies contributed to each pooled estimate. The present findings therefore indicate an association between the angiogenic profile before clinical recognition and the subsequent phenotype, rather than demonstrating clinical predictive utility.

### 3.4. Implications

The implications of these findings differ according to when measurement occurs. Because thresholds in current use were derived and validated in women with suspected PE [6,38], they should not be assumed to carry the same interpretation in normotensive FGR/SGA, and direct validation in that population is required.

The findings before clinical recognition raise a different possibility, which we state as a hypothesis for future research. Whether angiogenic markers measured before either condition is recognised can distinguish pregnancies that will subsequently develop PE-associated FGR/SGA from those that will subsequently develop isolated FGR/SGA has not been tested. Any clinical use for this purpose would require prospective evaluation with prespecified thresholds and clinically relevant performance measures, and the present findings do not support stratification in current practice.

A corresponding implication follows for research practice. PE-associated and normotensive impaired-growth pregnancies should not automatically be pooled in angiogenic biomarker research, because their average maternal angiogenic profiles differ. Studies of angiogenic markers in fetal growth restriction should report and stratify by maternal hypertensive status. Pooling the two phenotypes within a single endpoint may attribute to growth restriction an angiogenic signal that is partly driven by coexisting PE and may overstate the angiogenic disturbance attributable to normotensive growth restriction.

### 3.5. Strengths and Limitations

The principal strength is the comparator. Both groups had impaired fetal growth, so the comparison helps distinguish the additional angiogenic disturbance associated with maternal PE from that associated with impaired fetal growth without PE. Analyses were stratified by clinical sampling context throughout, and no pooled estimate combining the two contexts was interpreted.

Study quality was limited principally in the comparability domain: only 2 of 23 studies received both Newcastle–Ottawa comparability stars and 10 received neither, so control for gestational age at sampling and other potential confounders was generally inadequate. Residual confounding is therefore a central limitation of these findings rather than a peripheral one, and the pooled estimates should be read with that in mind.

Because maternal PlGF and sFlt-1 concentrations vary physiologically across pregnancy, differences in gestational age at blood sampling may confound comparisons between PE-associated and isolated FGR/SGA, and standardisation using Hedges’ g does not eliminate this potential bias. Sensitivity analyses restricted to studies reporting group-specific sampling gestational ages, including those with observed between-group differences of no more than 1.5 weeks, yielded broadly similar biomarker effects after clinical recognition. However, these findings do not exclude residual confounding by sampling gestational age. The small number of eligible studies, incomplete reporting, and imprecision of the more restrictive analysis before clinical recognition limit the conclusions that can be drawn.

Antenatally defined FGR and birthweight-defined SGA are not equivalent, and birthweight-defined SGA may include constitutionally small neonates in either comparison group. The extent and direction of any resulting bias cannot be determined from aggregate study-level data. Although definition-stratified analyses after clinical recognition showed consistent directions of effect across both strata, the combined estimates should not be interpreted as representing a single homogeneous growth-restriction phenotype.

Preeclampsia definitions changed over the period covered by this review. A definition requiring proteinuria and one admitting maternal organ dysfunction without proteinuria do not identify the same population, so the PE-associated groups pooled here were not assembled under a single diagnostic standard. This is a further source of clinical heterogeneity that cannot be resolved with aggregate data, and it compounds the heterogeneity in growth-outcome definition.

Heterogeneity was considerable for the sFlt-1/PlGF ratio and sFlt-1 after clinical recognition (I^2^ = 79.3% and 76.8%, respectively), so those pooled values should be read as averages across a varied evidence base rather than as uniform effects across studies. Exploratory moderator analyses identified three study-level associations—the FGR/SGA definition with the sFlt-1 effect after clinical recognition, assay class with the PlGF effect after clinical recognition, and gestational age at sampling with the ratio effect before clinical recognition. These associations were based on study-level data; several models contained few studies or limited variation in the moderator, and no adjustment was made for multiple testing; they are hypothesis-generating and are not interpreted as explanations of the pooled estimates.

Sampling context and growth-outcome definition were also structurally related, with seven of nine studies sampling before clinical recognition using birthweight-based outcomes and 11 of 14 studies sampling after clinical recognition using antenatal FGR definitions, so their independent contributions cannot be reliably separated, particularly in an analysis based on aggregate rather than individual participant data, and the observed between-context difference in sFlt-1 therefore cannot be attributed to the timing of clinical recognition alone, since it may also reflect the predominance of antenatally defined FGR among the studies sampling after clinical recognition. Gestational age at sampling and assay class may also differ across sampling contexts, further complicating the interpretation of between-context comparisons. Each feature was examined as far as aggregate data allow: pooled estimates stratified by growth-outcome definition, with a test between the strata (Supplementary Table S5); analyses restricted to studies reporting group-specific sampling gestational ages and to those with a between-group difference of no more than 1.5 weeks (Supplementary Table S6); and univariable meta-regression on each feature (Supplementary Table S3). These analyses preserved the direction of the pooled estimates and, with the exceptions reported above, their statistical significance; they cannot, however, separate the contribution of one feature from that of another, so how much of the between-context difference reflects the timing of sampling and how much reflects the differing composition of the contributing studies remains undetermined.

The three marker pools were not composed of identical studies and participants, so the magnitudes of their effect estimates were not formally compared. Several contributing studies had very small exposure groups, so individual study estimates were imprecise, and the pooled results are dominated by a few larger cohorts.

The analyses conducted after clinical recognition remained statistically significant across the other sensitivity analyses, whereas analyses restricted to directly reported means and standard deviations were imprecise and interpreted descriptively. Before clinical recognition, two analyses did not: sFlt-1 was no longer significant when Honigberg et al., 2018 [26] was omitted, and the sFlt-1/PlGF ratio was no longer significant when studies with fewer than 10 PE-associated cases were excluded; the PlGF analysis remained significant throughout, except in the restriction to studies with documented comparability of sampling gestational age, where three studies yielded an imprecise estimate (Section 2.8). Under Hartung–Knapp inference, all six analyses remained significant with wider intervals, with *p*-values of 0.026 to 0.044 before clinical recognition. Prediction intervals included the null in all six analyses, indicating that the effect observed in a new study could differ substantially from the pooled mean. Most effect estimates were reconstructed from medians and interquartile ranges, which is a further source of uncertainty, particularly for the potentially skewed sFlt-1/PlGF ratio. When analyses were restricted to directly reported means and standard deviations, the associations for sFlt-1 and the sFlt-1/PlGF ratio before clinical recognition were no longer statistically significant; these restricted analyses included only two studies each, so the effect of the reconstruction cannot be distinguished from differences in the studies included.

Egger tests were non-significant in all six analyses, but four contained fewer than ten studies and had limited power to detect asymmetry, so small-study effects cannot be excluded.

## 4. Materials and Methods

### 4.1. Protocol and Reporting

The protocol for this systematic review of maternal angiogenic markers in impaired fetal growth was prospectively registered in PROSPERO (CRD420261368726). This systematic review and meta-analysis was conducted and reported in accordance with the Preferred Reporting Items for Systematic Reviews and Meta-Analyses (PRISMA) 2020 statement [39]. The registered protocol prespecified two comparisons with different comparator groups, each reported in a separate manuscript. The companion analysis evaluates angiogenic biomarkers measured before clinical recognition in pregnancies that subsequently developed isolated FGR/SGA relative to unaffected pregnancies [40], whereas the present study compares maternal angiogenic profiles between PE-associated and isolated FGR/SGA. Because the two analyses use different comparator groups, no effect estimate or pooled result is shared between the two reports: six studies are common to both, and in each of these the isolated FGR/SGA group serves as the index group in the companion analysis and as the comparator here.

Stratification of the analyses by clinical sampling context and univariable meta-regression on study-level moderators were prespecified in the registered protocol. The sensitivity analyses reported in Section 2.8 and the pooled estimates stratified by growth-outcome definition were post hoc.

### 4.2. Eligibility Criteria

We included observational cohort and case–control studies of singleton pregnancies that measured maternal serum or plasma sFlt-1, PlGF, or the sFlt-1/PlGF ratio and allowed a direct comparison between PE-associated FGR/SGA and isolated FGR/SGA. PE and FGR/SGA were accepted as defined by each source study. The PE-associated group comprised pregnancies with both PE and FGR/SGA, whereas the isolated FGR/SGA group was required to be free of any hypertensive disorder of pregnancy.

For inclusion in the quantitative synthesis, studies were required to report group-specific sample sizes and summary statistics sufficient to calculate an effect size. Studies were excluded if PE could not be separated from a broader composite of hypertensive disorders, the normotensive status of the isolated FGR/SGA group could not be confirmed, the two comparison groups could not be extracted separately, or only a median with its confidence interval was reported, precluding recovery of a standard deviation. For overlapping cohorts, only the report with the most complete PE-stratified data was retained.

Because eligible studies used either antenatally defined FGR or birthweight-defined SGA, both were referred to collectively as FGR/SGA. Antenatally defined FGR referred to classifications based on fetal biometry and/or Doppler criteria, whereas SGA referred to classifications based on birthweight centiles. The detailed PE, FGR/SGA, and comparator definitions applied in each study are provided in Supplementary Table S1.

Within each study, both comparison groups met the same growth criterion, so the definition applied differs between studies rather than between the compared groups, and the pooled estimates combine within-study contrasts. The growth-outcome definition was nevertheless treated as a study-level characteristic: pooled estimates were also calculated separately for studies applying antenatally defined FGR and for those applying birthweight-defined SGA, and the definition was examined as a study-level moderator.

The composite was retained as an umbrella description of the growth-related outcomes applied in the source studies, and outcome definition was examined analytically rather than used as an inclusion criterion (Section 4.6).

### 4.3. Search Strategy and Study Selection

PubMed, Embase, and the Cochrane Central Register of Controlled Trials (CENTRAL) were searched from database inception through 13 April 2026. Controlled vocabulary and free-text terms for PlGF, sFlt-1 or soluble VEGFR-1, angiogenic ratios, and fetal growth outcomes, including FGR, IUGR, SGA, and placental insufficiency, were combined without date, language, or study-design restrictions. The reference lists of included studies and relevant systematic reviews were hand-searched; no additional eligible studies were identified. The complete search strategies as executed are provided in Supplementary File S1.

After duplicate records were removed, two reviewers independently screened titles and abstracts and assessed the full texts of potentially eligible articles. Disagreements were resolved through discussion. Reports arising from potentially overlapping cohorts were identified by comparing study centres, recruitment periods, participant characteristics, and sample sizes; where overlap was confirmed, a single report was retained according to the rule specified in Section 4.2. The study-selection process is presented in the PRISMA 2020 flow diagram (Figure 1).

### 4.4. Clinical Sampling Contexts

Studies were classified into two clinical sampling contexts. Sampling before clinical recognition comprised samples obtained before either PE or FGR/SGA was clinically recognised, with outcomes ascertained subsequently. Sampling after clinical recognition comprised samples obtained at or after the reported clinical diagnosis of PE and/or FGR/SGA. Because the timing of the PE diagnosis relative to recognition of FGR/SGA was not consistently reported, this category cannot be assumed to represent a clinically established maternal PE phenotype at the time of sampling; the clinical status of each comparison group at sampling is summarised for every study in Supplementary Table S1, with incompletely reported status identified as not confirmed. Each study contributed data to only one sampling context.

The two contexts were analysed and interpreted separately because maternal angiogenic profiles may differ according to when sampling occurs in relation to clinical recognition. Because the context-specific estimates were derived from different studies and participants, between-context differences were not interpreted as longitudinal biomarker changes.

The two contexts also differ in the growth-outcome definitions applied: seven of the nine studies sampling before clinical recognition used birthweight-defined SGA, whereas 11 of the 14 studies sampling after clinical recognition used antenatally defined FGR. Sampling context and growth-outcome definition are therefore partially confounded at the study level, so differences between the contexts cannot be attributed to the timing of sampling alone.

### 4.5. Data Extraction and Quality Assessment

Two reviewers independently extracted study design, cohort assembly, clinical status at the time of sampling, maternal age, PE and FGR/SGA definitions, group sizes, gestational age at sampling, assay platform, unit of measurement, and marker values. Study quality was assessed independently by two reviewers using the Newcastle–Ottawa Scale, with the cohort or case–control version selected according to the actual participant selection and sampling structure of each study rather than solely the design label used by its authors: the cohort version where participants were assembled as a single cohort and classified by phenotype thereafter, and the case–control version where the comparison groups were assembled on the basis of disease status and biomarker values were then ascertained. Comparability was specified before assessment: the first star required matching, statistical adjustment, or a protocol-fixed common sampling window for gestational age at sampling, for which matching on gestational age at delivery was not accepted, and the second star required matching or adjustment for maternal age, body mass index, parity or plurality. The version applied, and the rationale for its selection, are reported for every study in Supplementary Table S4. Discrepancies in data extraction and quality assessment were resolved through discussion.

Gestational age at blood sampling was extracted separately for the PE-associated and isolated FGR/SGA groups wherever reported. Information on matching or adjustment for gestational age at sampling and the use of gestational-age-standardised biomarker measurements was also recorded. When neither group-specific sampling gestational ages nor explicit evidence of between-group gestational-age matching or adjustment was available, between-group comparability of sampling gestational age was classified as not established.

When a study reported multiple eligible estimates from the same cohort, one estimate per marker and sampling context was selected to avoid double counting. Selection favoured, in order: (1) the most strictly defined PE-associated and normotensive isolated groups; (2) an antenatally defined FGR stratum rather than a stratum of small fetuses without additional Doppler or biometric criteria for FGR; (3) absolute concentrations rather than multiples of the median when the same participants were reported in both units; (4) directly measured free PlGF rather than total PlGF; and (5) when several gestational-age strata within the same sampling context remained eligible, the stratum with the largest PE-associated group or, if these were equal, the larger comparator group.

### 4.6. Statistical Analysis

When means and standard deviations were not reported, they were estimated from medians with the accompanying interquartile ranges using the methods proposed by Wan et al. [41]. Standard deviations were recovered from standard errors using SD = SE × √n. For each study, marker, and sampling context, the between-group effect was expressed as Hedges’ g, a standardised mean difference incorporating a small-sample correction. However, this standardisation does not adjust for potential differences in gestational age at blood sampling between the comparison groups. Positive values indicated higher marker levels in PE-associated FGR/SGA, whereas negative values indicated lower levels.

For each marker, pooled estimates were calculated using both fixed-effect and random-effects models. Because the included studies differed clinically and methodologically, random-effects estimates were used as the principal results throughout. Between-study variance was estimated using restricted maximum likelihood, and Wald-type confidence intervals were calculated for the pooled effect estimates. Statistical heterogeneity was assessed using Cochran’s Q test and quantified using the I^2^ statistic.

Separate pooled analyses were performed for the sFlt-1/PlGF ratio, sFlt-1, and PlGF within the sampling contexts before and after clinical recognition. Differences in effect estimates between the two contexts were examined as exploratory analyses using tests for subgroup differences and meta-regression with sampling context as a study-level moderator. The subgroup-difference test estimated between-study variance separately within each sampling context, whereas meta-regression assumed a common between-study variance. No adjustment for multiplicity was applied to the six pooled analyses. Pooled estimates were interpreted by their magnitude and the width of their confidence interval rather than by statistical significance alone, and analyses to which few studies contributed were treated as imprecise even where the interval excluded the null.

Within each sampling context, exploratory univariable meta-regression analyses examined whether effect estimates were associated with maternal age, gestational age at sampling, FGR/SGA definition, assay class, or clinical status at the time of sampling (unselected, high-risk or suspected, or confirmed disease). Studies without an estimable covariate were excluded from the corresponding model. A moderator was not analysed when the number of studies was insufficient, or there was inadequate between-study variation for estimation. Twenty-five of the 30 possible marker–moderator combinations were estimable. No adjustment for multiplicity was applied to these models, and with this number of tests and few studies per model, nominally significant associations may arise by chance; all moderator findings are therefore reported as hypothesis-generating and are not used to support mechanistic conclusions.

Pooled estimates were additionally calculated separately for studies applying antenatally defined FGR and for those applying birthweight-defined SGA within each sampling context, using the same random-effects specification; strata of fewer than two studies were not pooled.

To assess potential confounding by gestational age at blood sampling, complementary sensitivity analyses were conducted. These included restriction to studies reporting group-specific sampling gestational ages; a post hoc exploratory restriction to studies with an absolute between-group sampling gestational-age difference of no more than 1.5 weeks; and restriction to studies with documented between-group comparability of sampling gestational age or gestational-age-standardised biomarker measurements. Exploratory meta-regression examined the association between the study-level difference in sampling gestational age and biomarker effect size where sufficient data were available. These analyses were added after protocol registration in response to peer review and are reported as post hoc sensitivity analyses.

Several further analyses were performed to examine the robustness of the pooled estimates. Hartung–Knapp confidence intervals [42] were calculated alongside the primary restricted maximum likelihood estimates with Wald-type intervals, and 95% prediction intervals [43] were calculated for each pooled analysis. Influence was examined by omitting each study in turn. Further analyses excluded studies contributing fewer than 10 PE-associated cases, studies with lower Newcastle–Ottawa ratings, and case–control designs, and restricted each analysis to effect estimates derived from directly reported means and standard deviations. These analyses were added after protocol registration in response to peer review and are reported as post hoc sensitivity analyses.

Funnel plots and Egger’s regression tests were used to explore small-study effects. All tests were two-sided, and *p* < 0.05 was considered statistically significant. Analyses were performed using R version 4.6.0 (R Foundation for Statistical Computing, Vienna, Austria) with the meta (version 8.5-0) and metafor (version 5.0-1) packages. The complete analysis data set is provided in Supplementary Table S2.

## 5. Conclusions

PE-associated and isolated FGR/SGA differed consistently in their maternal angiogenic profiles, with higher sFlt-1, lower PlGF and a higher sFlt-1/PlGF ratio in PE-associated pregnancies. Differences in the same directions were present in studies that sampled before either condition was clinically recognised, and after clinical recognition they were also seen when analyses were restricted to studies applying antenatal FGR criteria. Because both comparison groups had impaired fetal growth, the differences observed may more specifically reflect angiogenic alterations associated with maternal PE, although residual confounding cannot be excluded. The PlGF difference was of similar magnitude in the two sampling contexts, whereas the sFlt-1 difference was larger after clinical recognition; this contrast was not consistently supported across analytical approaches and is therefore reported as an exploratory comparison between studies rather than as evidence of change over time within pregnancies.

Interpretation is limited by substantial between-study heterogeneity in some analyses and by heterogeneity in the FGR/SGA definitions applied, in gestational age at blood sampling and in assay characteristics. Control for gestational age at sampling and other potential confounders was incomplete in most contributing studies, so residual confounding is a central rather than a peripheral limitation. These are group-level differences in biomarker distributions and do not establish individual-level prediction, discrimination, threshold performance or incremental clinical value.

The principal implication is for research practice: PE-associated and normotensive impaired-growth pregnancies should not automatically be pooled in angiogenic biomarker research, because their average maternal angiogenic profiles differ. Angiogenic thresholds derived in suspected PE require direct validation before being applied to normotensive FGR/SGA.

## Figures and Tables

**Table 1:** Characteristics of the 23 studies included in the pooled analyses.

Study	Design	Cohort Assembly	Growth Phenotype	Sampling Context (GA)	PE-FGR (*n*)	Isolated FGR (*n*)	Markers
Shibata, 2005 [16]	Case–control	Unselected	SGA	After recognition, GA NR	5	22	sFlt-1, PlGF
Savvidou, 2006 [17]	Prospective cohort	High-risk/suspected	FGR	After recognition, 23–25 wk	3	12	sFlt-1
Wathén, 2006 [18]	Case–control	Unselected	SGA	Before recognition, 16–20 wk	8	10	sFlt-1
Diab, 2008 [19]	Prospective cohort	High-risk/suspected	FGR	Before recognition, 23 wk	8	9	Ratio, sFlt-1, PlGF
Savvidou, 2008 [20]	Prospective cohort	High-risk/suspected	SGA	Before recognition, 23–25 wk	5	15	Ratio, PlGF
Cowans, 2010 [21]	Case–control	Confirmed disease	SGA	Before recognition, 11+0–13+6 wk	7	8	PlGF
Ghosh, 2013 [22]	Prospective cohort	Unselected	FGR	Before recognition, 20–22 wk	11	21	PlGF
Alahakoon, 2014 [23]	Prospective case–control	Confirmed disease	FGR	After recognition, 24–41 wk	16	24	Ratio, sFlt-1, PlGF
Herraiz, 2014 [14]	Case–control	Confirmed disease	FGR	After recognition, 24+0–33+6 wk	22	19	Ratio, sFlt-1, PlGF
Laskowska, 2015 [24]	Case–control	Confirmed disease	FGR	After recognition, 2nd–3rd trimester	64	65	sFlt-1 (sVEGFR-1)
Kienast, 2016 [25]	Prospective cohort	High-risk/suspected	SGA	Before recognition, 18–25 wk	14	26	Ratio, sFlt-1, PlGF
Honigberg, 2018 [26]	Prospective cohort	Unselected	SGA	Before recognition, 26 wk	47	238	Ratio, sFlt-1, PlGF
Fabjan-Vodusek, 2019 [27]	Prospective cross-sectional	Confirmed disease	FGR	After recognition, ≥24 wk	32	12	Ratio, sFlt-1, PlGF
Zhang, 2019 [28]	Prospective cohort	Unselected	SGA	Before recognition, 11+0–13+6 wk	15	84	PlGF
Schoots, 2021 [15]	Prospective clinical pilot	Confirmed disease	FGR	After recognition, 24–36 wk	11	14	Ratio, sFlt-1, PlGF
Lorenz-Meyer, 2022 [29]	Retrospective cohort	High-risk/suspected	FGR	After recognition, 3rd trimester	25	40	Ratio, sFlt-1, PlGF
Dymara-Konopka, 2023 [13]	Prospective case–control	Confirmed disease	FGR	After recognition, close to delivery	22	14	Ratio, sFlt-1, PlGF
Kluivers, 2023 [30]	Prospective cohort	High-risk/suspected	SGA	After recognition, <34+0 wk	23	9	Ratio, sFlt-1, PlGF
Rajiv, 2024 [31]	Case–control	Confirmed disease	FGR	After recognition, mean 33.5 wk	82	39	sFlt-1, PlGF
Zhang, 2024 [32]	Case–control	Confirmed disease	SGA	Before recognition, 2nd trimester	40	186	PlGF
Jungelson, 2025 [33]	Retrospective cohort	High-risk/suspected	SGA	After recognition, 28+0–33+6 wk	25	42	Ratio, sFlt-1, PlGF
Shinohara, 2026 [34]	Retrospective cohort	Confirmed disease	FGR	After recognition, 22+0–33+6 wk	65	267	PlGF
Youssef, 2026 [35]	Prospective cohort	Confirmed disease	FGR	After recognition, ≥28+0 wk	80	300	Ratio, sFlt-1, PlGF

GA, gestational age; PE, preeclampsia; FGR, fetal growth restriction; SGA, small for gestational age; sFlt-1, soluble fms-like tyrosine kinase-1; PlGF, placental growth factor; sVEGFR-1, soluble vascular endothelial growth factor receptor-1 (synonymous with sFlt-1); Ratio, sFlt-1/PlGF ratio; NR, not reported. Cohort assembly describes how participants were identified for the study: unselected = general antenatal screening population; high-risk/suspected = selected because of abnormal uterine artery Doppler or clinical suspicion; confirmed disease = selected on the basis of an established diagnosis of FGR and/or PE, including studies in which cases were identified retrospectively after delivery. Sampling context refers to when blood was sampled in relation to clinical recognition: before recognition = sampled before either PE or FGR/SGA was clinically recognised; after recognition = sampled at or after clinical diagnosis.

**Table 2:** Comparison of between-phenotype effect estimates across sampling contexts.

Marker	Sampling Before Clinical Recognition Hedges’ g (95% CI) [95% PI]	Sampling After Clinical Recognition Hedges’ g (95% CI) [95% PI]	Subgroup Difference $\chi_{1}^{2}$	*p*	Meta-Regression B (95% CI)	*p*
sFlt-1/PlGF ratio	0.86 (0.38 to 1.33) [−0.89 to 2.60]	1.04 (0.51 to 1.57) [−0.81 to 2.88]	0.25	0.618	0.19 (−0.66 to 1.03)	0.666
sFlt-1	0.38 (0.12 to 0.64) [−0.20 to 0.96]	1.05 (0.73 to 1.38) [−0.08 to 2.19]	10.06	0.002	0.60 (−0.03 to 1.23)	0.064
PlGF	−0.46 (−0.77 to −0.16) [−1.27 to 0.34]	−0.60 (−0.82 to −0.38) [−1.25 to 0.05]	0.50	0.479	−0.13 (−0.50 to 0.24)	0.488

CI, confidence interval; PI, prediction interval; PlGF, placental growth factor; sFlt-1, soluble fms-like tyrosine kinase-1. B represents the difference in Hedges’ g for sampling after clinical recognition relative to sampling before it. Values in square brackets are 95% prediction intervals, which estimate the range within which the effect in a new study would be expected to lie and therefore reflect between-study heterogeneity in addition to the uncertainty of the pooled mean. The test for subgroup differences estimates between-study variance separately within each sampling context, whereas meta-regression assumes a common between-study variance; the two results are therefore not interchangeable, and both are reported. These between-context comparisons were considered exploratory.

## Data Availability

The original contributions presented in this study are included in the article/Supplementary Material. Further inquiries can be directed to the corresponding authors.

## Supplementary Materials

The following supporting information can be downloaded at: https://www.mdpi.com/article/10.3390/ijms27219829/s1.
