# Supplementary material for: Maternal Angiogenic Profiles in Preeclampsia-Associated Versus Isolated Fetal Growth Restriction or Small-for-Gestational-Age Pregnancies: A Systematic Review and Meta-Analysis
Source: Int J Mol Sci. 2026 Nov 2;27(21):9829. doi: 10.3390/ijms27219829 (PMC13763136; doi:10.3390/ijms27219829)
Supplement: Supplementary file 1 [file ijms-27-09829-s001.zip › ijms-4590317-supplementary.pdf]

Supplementary Materials

Maternal Angiogenic Profiles in Preeclampsia-Associated versus Isolated Fetal Growth Restriction or Small-for-Gestational-Age Pregnancies: A Systematic Review and Meta-Analysis

Contents

File S1. Complete search strategies

Table S1. Study-level characteristics central to the interpretation of the pooled comparisons (S1a, definitions and their classification; S1b, clinical status and gestational age at sampling)

Table S2. Analysis data set

Table S3. Exploratory univariable meta-regression on study-level moderators, by clinical sampling context

Table S4. Newcastle–Ottawa Scale item-level ratings for the 23 included studies, with the scale version applied to each study

Table S5. Pooled estimates stratified by growth-outcome definition, by clinical sampling context

Table S6. Results of the sensitivity analyses for the six pooled comparisons

Figure S1. Funnel plots for the six pooled analyses

File S1. Complete search strategies

Searches were executed on 13 April 2026.

PubMed/MEDLINE

No.	Query	Results
#6	"Placenta Growth Factor"[Mesh] OR "Vascular Endothelial Growth Factor Receptor-1"[Mesh]	6757
#7	"PlGF"[tiab] OR "placenta growth factor"[tiab] OR "placental growth factor"[tiab] OR "sFlt-1"[tiab] OR "sFlt1"[tiab] OR "soluble fms-like tyrosine kinase"[tiab] OR "soluble fms like tyrosine kinase"[tiab] OR "sVEGFR-1"[tiab] OR "sVEGFR1"[tiab] OR "angiogenic factor*"[tiab] OR "antiangiogenic factor*"[tiab] OR "sFlt-1/PlGF"[tiab] OR "PlGF/sFlt-1"[tiab]	17882
#8	#6 OR #7	20492
#9	"Fetal Growth Retardation"[Mesh]	20369
#10	"IUGR"[tiab] OR "intrauterine growth restriction"[tiab] OR "intrauterine growth retardation"[tiab] OR "fetal growth restriction"[tiab] OR "fetal growth retardation"[tiab] OR "foetal growth restriction"[tiab] OR "FGR"[tiab] OR "small for gestational age"[tiab] OR "SGA"[tiab] OR "placental insufficiency"[tiab] OR "placental dysfunction"[tiab]	46311
#11	#9 OR #10	52634
#12	#8 AND #11	1106

Embase

No.	Query	Results
#1	'placental growth factor'/exp OR 'vasculotropin receptor 1'/exp	19256
#2	'plgf':ti,ab OR 'placenta* growth factor':ti,ab OR 'sflt-1':ti,ab OR 'sflt1':ti,ab OR 'vasculotropin receptor 1':ti,ab OR 'soluble fms-like tyrosine kinase':ti,ab OR 'soluble fms like tyrosine kinase':ti,ab OR 'svegfr-1':ti,ab OR 'svegfr1':ti,ab OR 'angiogenic factor*':ti,ab OR 'antiangiogenic factor*':ti,ab OR 'sflt-1/plgf':ti,ab	26491
#3	#1 OR #2	36980
#4	'intrauterine growth retardation'/exp OR 'placental insufficiency'/exp	72214
#5	'iugr':ti,ab OR 'intrauterine growth restriction':ti,ab OR 'intrauterine growth retardation':ti,ab OR 'fetal growth restriction':ti,ab OR 'fetal growth retardation':ti,ab OR 'foetal growth restriction':ti,ab OR 'fgr':ti,ab OR 'small for gestational age':ti,ab OR 'sga':ti,ab OR 'placental insufficiency':ti,ab OR 'placental dysfunction':ti,ab	71026
#6	#4 OR #5	97850
#7	#3 AND #6	2372

Cochrane Library (CENTRAL)

ID	Search	Results
#1	MeSH descriptor: [Placenta Growth Factor] explode all trees	120
#2	MeSH descriptor: [Vascular Endothelial Growth Factor Receptor-1] explode all trees	120
#3	(PlGF OR "placenta growth factor" OR "placental growth factor"):ti,ab,kw	461
#4	(sFlt-1 OR sFlt1 OR "soluble fms-like tyrosine kinase" OR "soluble fms like tyrosine kinase" OR sVEGFR-1 OR sVEGFR1 OR "soluble flt-1" OR "soluble flt1" OR "VEGFR-1" OR "FLT1"):ti,ab,kw	444
#5	#1 OR #2 OR #3 OR #4	742
#6	MeSH descriptor: [Fetal Growth Retardation] explode all trees	636
#7	(IUGR OR "intrauterine growth restriction" OR "intrauterine growth retardation" OR "fetal growth restriction" OR "fetal growth retardation" OR "foetal growth restriction" OR FGR OR "small for gestational age" OR SGA OR "placental insufficiency" OR "placental dysfunction"):ti,ab,kw	4282
#8	#6 OR #7	4282
#9	#5 AND #8	102

Supplementary Table S1a. Preeclampsia, FGR/SGA and comparator definitions applied in each included study, with the classification of each definition.

Study	Preeclampsia definition	PE definition classification	FGR / SGA definition	Growth outcome classification	Isolated (normotensive) comparator definition
Shibata 2005 [16]	Gestational hypertension with proteinuria and hyperuricaemia, with reversal of hypertension and proteinuria after delivery; proteinuria >300 mg/24 h, >2+ on a voided sample, or protein/creatinine ratio >0.3.	Proteinuria required	SGA, birthweight <10th centile.	Birthweight-defined SGA	SGA without PE.
Savvidou 2006 [17]	ISSHP — diastolic blood pressure ≥110 mmHg on one occasion or ≥90 mmHg on two consecutive occasions >4 h apart, with proteinuria (≥300 mg/24 h or ≥2+ on dipstick on two consecutive occasions) developing after 20 weeks in a previously normotensive woman.	Proteinuria required	FGR, estimated fetal weight <5th centile at recruitment and birthweight <5th centile (sex-adjusted); 11 of 15 had absent or reversed umbilical artery end-diastolic flow.	Antenatally defined FGR	FGR in women who did not develop PE.
Wathen 2006 [18]	Blood pressure >140/90 mmHg measured at least twice 24 h apart with proteinuria >0.3 g/24 h; severe PE was blood pressure >160/110 mmHg and/or proteinuria >5 g/24 h.	Proteinuria required	IUGR defined as a birth weight at least 2 SD below the national average for gestational age.	Birthweight-defined SGA	IUGR without PE.
Diab 2008 [19]	New-onset hypertension and proteinuria after 20 weeks, proteinuria being protein excretion of at least 300 mg in a 24-h urine specimen with no evidence of urinary tract infection.	Proteinuria required	IUGR defined as an estimated fetal weight below the 5th percentile of an ultrasound biometry reference.	Antenatally defined FGR	IUGR without maternal hypertension
Savvidou 2008 [20]	ISSHP — diastolic blood pressure ≥110 mmHg on one occasion or ≥90 mmHg on two consecutive occasions >4 h apart, with proteinuria (≥300 mg/24 h or ≥2+ on dipstick) developing after 20 weeks in a previously normotensive woman.	Proteinuria required	SGA by birthweight; centile not specified.	Birthweight-defined SGA	SGA without PE.
Cowans 2010 [21]	ISSHP (Davey and MacGillivray 1988).	Proteinuria required	SGA, birthweight <10th centile.	Birthweight-defined SGA	SGA without PE.
Ghosh 2013 [22]	ISSHP definition as set out by Davey and MacGillivray (1988), the reference cited by the source, which requires proteinuria; the criteria were not restated in the source.	Proteinuria required	Antenatally defined FGR based on ultrasound biometry and umbilical artery Doppler assessment at 32 weeks.	Antenatally defined FGR	IUGR without PE.
Alahakoon 2014 [23]	ISSHP — diastolic blood pressure ≥90 mmHg on two occasions ≥4 h apart with proteinuria >300 mg/24 h.	Proteinuria required	IUGR, birthweight <10th centile with umbilical artery S/D ratio or resistance index >95th centile.	Antenatally defined FGR	IUGR without PE.
Herraiz 2014 [14]	NHBPEP Working Group criteria; HELLP by Sibai criteria; superimposed PE as pre-existing hypertension before 20 weeks with de-novo proteinuria or a sudden increase in hypertension. PE, HELLP and superimposed PE were combined into a single hypertensive group.	Organ dysfunction accepted without proteinuria	FGR, Hadlock estimated fetal weight <10th centile with amniotic fluid index <10th centile or umbilical artery PI >95th centile; SGA fetuses not meeting FGR criteria were excluded.	Antenatally defined FGR	FGR in normotensive women.
Laskowska 2015 [24]	Severe PE — blood pressure >160/110 mmHg on two occasions 6 h apart, proteinuria >5 g/24 h, and at least one of renal or haematological abnormality, HELLP, or neurological symptoms; normotensive before 20 weeks, without chronic hypertension or renal disease.	Proteinuria required	IUGR, fetal weight below the 10th centile for gestational age on ultrasound (Polish population curves) with at least one marker of disturbed placental function, confirmed by birthweight.	Antenatally defined FGR	IUGR in normotensive women.
Kienast 2016 [25]	New-onset proteinuric hypertension after 20 weeks; hypertension as systolic ≥140 mmHg or diastolic ≥90 mmHg, proteinuria as at least 1+ on dipstick in the absence of urinary tract infection.	Proteinuria required	FGR defined as a birth weight below the 10th percentile for gestational age.	Birthweight-defined SGA	FGR without PE.
Honigberg 2018 [26]	Systolic blood pressure ≥140 and/or diastolic ≥90 mmHg from the second study visit (18 weeks) onward with urine protein/creatinine ratio >0.2 and/or >300 mg protein on 24-h collection; hypertensive pregnancies were adjudicated by a panel of investigators.	Proteinuria required	Birthweight Z-score < −1.0 (approximately the 16th centile); Z < −2.0 was available as a stricter alternative.	Birthweight-defined SGA	Birthweight Z-score < −1.0 without PE.
Fabjan-Vodušek 2019 [27]	ACOG 2013 Task Force on Hypertension in Pregnancy.	Organ dysfunction accepted without proteinuria	FGR, estimated fetal weight or abdominal circumference <5th centile with oligohydramnios (amniotic fluid index <5 cm) and/or umbilical artery PI >95th centile.	Antenatally defined FGR	FGR without PE.
Zhang 2019 [28]	New-onset hypertension (≥140 mmHg systolic and/or ≥90 mmHg diastolic on ≥2 occasions 4 h apart) after 20 weeks together with proteinuria (≥300 mg/24 h or protein/creatinine ratio ≥30 mg/mmol).	Proteinuria required	SGA by birthweight; centile not specified in the source.	Birthweight-defined SGA	SGA without PE.
Schoots 2021 [15]	Criteria not reported; the source defines hypertension (systolic >140 and diastolic >90 mmHg) only in relation to the comparator group.	Not reported in sufficient detail to classify	Severe FGR — abdominal circumference or estimated fetal weight <3rd centile, or absent umbilical artery end-diastolic flow.	Antenatally defined FGR	Severe FGR without PE.

Lorenz-Meyer 2022 [29]	ISSHP 2014 and the German obstetric guideline — systolic ≥140 or diastolic ≥90 mmHg on two occasions ≥4 h apart in a previously normotensive woman, with proteinuria (dipstick 2+ twice or ≥300 mg/24 h).	Proteinuria required	IUGR, estimated fetal weight <10th centile and/or an inadequate growth trajectory with pathological umbilical or uterine artery Doppler or oligohydramnios.	Antenatally defined FGR	IUGR without a hypertensive disorder of pregnancy.
Dymara-Konopka 2023 [13]	ISSHP 2018 — new-onset hypertension (≥140 mmHg systolic or ≥90 mmHg diastolic) on two or more consecutive occasions with new-onset proteinuria (>0.3 g/24 h) or, in the absence of proteinuria, other maternal organ or uteroplacental dysfunction.	Organ dysfunction accepted without proteinuria	FGR — abnormal Doppler (uterine or umbilical artery PI >95th centile, or middle cerebral artery or cerebroplacental ratio <5th centile) in 30 of 36, or estimated fetal weight <3rd centile alone in 6 of 36.	Antenatally defined FGR	Normotensive FGR
Kluivers 2023 [30]	De-novo hypertension (systolic ≥140 and diastolic ≥90 mmHg) with proteinuria (uPCR ≥30 mg/mmol or ≥0.3 g/24 h), superimposed PE, or (partial) HELLP syndrome.	Organ dysfunction accepted without proteinuria	SGA, birthweight <10th centile. The authors state that SGA was used as a proxy for FGR because biometry and Doppler data were not available.	Birthweight-defined SGA	SGA without PE.
Rajiv 2024 [31]	ACOG — de novo hypertension at or after 20 weeks (systolic ≥140 or diastolic ≥90 mmHg) with end-organ dysfunction	Organ dysfunction accepted without proteinuria	FGR — birthweight ≤10th centile with at least one of umbilical artery PI >95th centile or absent/reversed end-diastolic flow, middle cerebral artery PI <5th centile, amniotic fluid index <6 cm, or cerebroplacental ratio <1.0.	Antenatally defined FGR	FGR without PE.
Zhang 2024 [32]	Clinical PE; criteria not reported — comorbidities including preeclampsia were taken from the mothers' medical records.	Not reported in sufficient detail to classify	SGA, birthweight <10th centile for gestational age (WHO 1995).	Birthweight-defined SGA	SGA without PE.
Jungelson 2025 [33]	Systolic ≥140 mmHg and/or diastolic ≥90 mmHg in a woman normotensive before 20 weeks with significant proteinuria (≥300 mg/24 h or protein/creatinine ratio >0.3); in chronic hypertension, superimposed PE by new-onset proteinuria, or by end-organ damage where proteinuria pre-existed.	Proteinuria required	SGA, birthweight ≤10th centile; Doppler criteria were not used.	Birthweight-defined SGA	SGA without PE.
Shinohara 2026 [34]	ISSHP 2021 clinical criteria	Organ dysfunction accepted without proteinuria	FGR, ultrasound estimated fetal weight below mean −1.5 SD (approximately the 6.7th centile, JSOG standard); severe FGR <3rd centile.	Antenatally defined FGR	Early-onset FGR without a hypertensive disorder of pregnancy.
Youssef 2026 [35]	Blood pressure ≥140/90 mmHg on two occasions ≥4 h apart after 20 weeks with proteinuria ≥300 mg/24 h or protein/creatinine ratio ≥0.3.	Proteinuria required	FGR, estimated fetal weight <10th centile with cerebroplacental ratio <5th centile, uterine artery PI >95th centile, or birthweight <3rd centile.	Antenatally defined FGR	FGR without PE.

PE, preeclampsia; FGR, fetal growth restriction; IUGR, intrauterine growth restriction; SGA, small for gestational age; ACOG, American College of Obstetricians and Gynecologists; ISSHP, International Society for the Study of Hypertension in Pregnancy; JSOG, Japan Society of Obstetrics and Gynecology; NHBPEP, National High Blood Pressure Education Program; PI, pulsatility index; SD, standard deviation.

Definitions are reproduced as reported by the source studies. The preeclampsia definition was classified as requiring proteinuria (15 studies), accepting maternal organ dysfunction without proteinuria (6), or not reported in sufficient detail to classify (2). Studies that did not state the criterion were retained provided that the two comparison groups could be identified separately and the comparator was described as free of hypertensive disease. The growth outcome was classified as antenatally defined FGR (13 studies) or birthweight-defined SGA (10).

Supplementary Table S1b. Clinical status at the time of blood sampling, gestational age at sampling, and control for gestational age at sampling in each included study.

Study	Clinical status at the time of blood sampling	GA at sampling, PE-associated	GA at sampling, isolated FGR/SGA	Difference (wk)	Matching or adjustment for GA at sampling	GA-standardised values
Shibata 2005 [16]	PE recognised; FGR/SGA status at sampling not confirmed	PE+SGA subgroup NR; overall PE 35.2±3.8	Overall SGA 39.1±1.6	Not calculable	Adjusted for GA at sampling (with labour, smoking, pre-pregnancy BMI) in a model reporting this contrast	No
Savvidou 2006 [17]	FGR/SGA recognised; PE status at sampling not confirmed	23–25	23–25	Not calculable	Protocol-fixed common window (23–25 wk); no further matching or adjustment	No
Wathen 2006 [18]	Sampled before clinical recognition	16–20	16–20	Not calculable	Protocol-fixed screening visits (13.7 ± 0.5 and 19.2 ± 0.6 wk); no further matching or adjustment	No
Diab 2008 [19]	Sampled before clinical recognition	23	23	Not calculable	Protocol-fixed single visit (23 wk); BMI adjustment applied to a different comparison	No
Savvidou 2008 [20]	Sampled before clinical recognition	23–25	23–25	Not calculable	Protocol-fixed common window (23–25 wk); no further matching or adjustment	No
Cowans 2010 [21]	Sampled before clinical recognition	11.0–13.9	11.0–13.9	Not calculable	Protocol-fixed window (11+0–13+6 wk); controls matched for GA (± 4 d) and storage time	MoM (gestation-specific median)
Ghosh 2013 [22]	Sampled before clinical recognition	20–22	20–22	Not calculable	Protocol-fixed common window (20–22 wk); no further matching or adjustment	No

Alahakoon 2014 [23]	Both conditions recognised	32.9 (28.2–34.6)	35.5 (31.3–37.0)	−2.6	None for GA at sampling (blood drawn within 24 h before delivery); adjusted for maternal age and parity	No
Herraiz 2014 [14]	Both conditions recognised	29.3±3.2	28.1±2.5	+1.2	Pairwise GA matching (± 3 d) to healthy controls only; compared groups analysed within GA phases	No
Laskowska 2015 [24]	Both conditions recognised	NR	NR	Not calculable	None; GA at sampling not reported	No
Kienast 2016 [25]	Sampled before clinical recognition	22 (18–25)	21 (19–24)	+1.0	Protocol-fixed visit window (18–25 wk); no further matching or adjustment	No
Honigberg 2018 [26]	Sampled before clinical recognition	NR	NR	Not calculable	Protocol third study visit for all participants (median 26.0 wk)	No
Fabjan-Vodušek 2019 [27]	Both conditions recognised	<34 wk 23/32 (72%); ≥34 wk 9/32 (28%)	<34 wk 9/12 (75%); ≥34 wk 3/12 (25%)	Not calculable	None; sampling from ≥ 24 wk with no common window	No
Zhang 2019 [28]	Sampled before clinical recognition	11.0–13.9	11.0–13.9	Not calculable	Protocol-fixed first-trimester window (11+0–13+6 wk); additionally adjusted for maternal weight, ethnicity and smoking	MoM (by gestational day)
Schoots 2021 [15]	Both conditions recognised	29.4	33.4	−4.0	None; inclusion window 24–36 wk	No
Lorenz-Meyer 2022 [29]	Both conditions recognised	32.6 (28.6-34.0)	33.4 (31.6-36.3)	−0.8	None; multivariable models addressed placental weight, not this contrast	No
Dymara-Konopka 2023 [13]	Both conditions recognised	32 (28–34)	35 (33–37)	−3.0	Pairwise GA matching to healthy controls only; none between the compared groups	No
Kluivers 2023 [30]	PE recognised; FGR/SGA status at sampling not confirmed	28 (24–31)	27 (24–31)	+1.0	None; analysis stratified by onset < 34 vs ≥ 34 wk only	No
Rajiv 2024 [31]	Both conditions recognised	NR	NR	Not calculable	None for GA at sampling; model adjusted for PE as a confounder	No
Zhang 2024 [32]	Sampled before clinical recognition	14–20	14–20	Not calculable	Protocol-fixed second-trimester monitoring window (14–20 wk); the 1:1 matching was on GA at birth	No
Jungelson 2025 [33]	PE recognised; FGR/SGA status at sampling not confirmed	32.5 [29.4–33.8]	31.3 [29.4–32.7]	+1.2	None for this contrast; total PlGF presented in sampling-GA bands descriptively	No
Shinohara 2026 [34]	FGR/SGA recognised; PE status at sampling not confirmed	22.0–33.9	22.0–33.9	Not calculable	None; sampling on the day of early-onset FGR diagnosis	No
Youssef 2026 [35]	Mixed within the study population	NR	NR	Not calculable	No matching or additional adjustment beyond GA-adjusted MoM values	MoM (GA-adjusted)

GA, gestational age; MoM, multiples of the median; NR, not reported; PE, preeclampsia.

Clinical status at the time of blood sampling: both conditions recognised = both PE and FGR/SGA had been clinically recognised when the sample was obtained; PE recognised, FGR/SGA status not confirmed, and FGR/SGA recognised, PE status not confirmed = one condition had been recognised and the source report does not establish whether the other had been diagnosed, which indicates unconfirmed rather than absent disease; mixed within the study population = the sampled participants were heterogeneous in this respect; sampled before clinical recognition = the sample preceded clinical recognition of either condition. No study was indeterminate for both conditions.

Gestational ages are reproduced as reported by each source study. Where the two sampling-GA columns carry the same entry, the study reported only a common sampling window for the whole study population and no group-specific values; the between-group difference is then not calculable. Group-specific values were reported by eight studies, a common window only by nine, partial information by two (Fabjan-Vodušek 2019 [27], Shibata 2005 [16]), and no sampling gestational age by four (Youssef 2026 [35], Honigberg 2018 [26], Laskowska 2015 [24], Rajiv 2024 [31]).

The difference is calculated as PE-associated minus isolated FGR/SGA, so a negative value indicates earlier sampling in the PE-associated group.

Biomarker values were entered into the analyses as multiples of the median in two studies (Cowans 2010 [21] and Youssef 2026 [35]) and as absolute concentrations in the remaining 21.

Two studies (Herraiz 2014 [14]; Kluivers 2023 [30]) reported separate strata below and at or beyond 34 weeks at diagnosis or enrolment. The below-34-week strata were used in the primary analyses because combined data were not reported. Combining both strata changed the pooled estimates after clinical recognition by less than 0.05.

Supplementary Table S2. Analysis data set

Study	Marker	Sampling context	Gestational age at sampling (as reported)	n PE / n isolated	PE-associated FGR/SGA, mean (SD)	Isolated FGR/SGA, mean (SD)	Unit	Hedges' g	SE
Alahakoon 2014 [23]	sFlt-1/PlGF ratio	After clinical recognition	24-41 wk	16 / 24	544 (780)	508 (734)	ratio	0.05	0.316
Herraiz 2014 [14]	sFlt-1/PlGF ratio	After clinical recognition	24+0-33+6 wk	22 / 19	485 (356)	108 (128)	ratio	1.34	0.343
Fabjan-Vodušek 2019 [27]	sFlt-1/PlGF ratio	After clinical recognition	>=24 wk	32 / 12	324 (334)	189 (246)	ratio	0.42	0.336
Schoots 2021 [15]	sFlt-1/PlGF ratio	After clinical recognition	24-36 wk	11 / 14	1,362 (586)	54.7 (79.7)	ratio	3.23	0.616
Lorenz-Meyer 2022 [29]	sFlt-1/PlGF ratio	After clinical recognition	3rd trimester	25 / 40	303 (295)	154 (186)	ratio	0.63	0.258
Dymara-Konopka 2023 [13]	sFlt-1/PlGF ratio	After clinical recognition	close to delivery	22 / 14	2,577 (4,638)	408 (438)	ratio	0.58	0.342
Kluivers 2023 [30]	sFlt-1/PlGF ratio	After clinical recognition	<34+0 wk	23 / 9	543 (759)	67.3 (163)	ratio	0.71	0.394
Jungelson 2025 [33]	sFlt-1/PlGF ratio	After clinical recognition	28+0-33+6 wk	25 / 42	237 (186)	12.3 (20.7)	ratio	1.94	0.302
Youssef 2026 [35]	sFlt-1/PlGF ratio	After clinical recognition	>=28+0 wk	80 / 300	10.8 (11.7)	3.34 (4.74)	MoM	1.09	0.132
Shibata 2005 [16]	sFlt-1	After clinical recognition	At diagnosis	5 / 22	7,660 (4,059)	2,307 (1,629)	pg/mL	2.35	0.584
Savvidou 2006 [17]	sFlt-1	After clinical recognition	23-25 wk	3 / 12	2,697 (1,015)	1,479 (892)	pg/mL	1.26	0.656
Alahakoon 2014 [23]	sFlt-1	After clinical recognition	24-41 wk	16 / 24	8,938 (381)	7,655 (3,345)	pg/mL	0.48	0.321
Herraiz 2014 [14]	sFlt-1	After clinical recognition	24+0-33+6 wk	22 / 19	13,524 (9,850)	6,497 (5,323)	pg/mL	0.85	0.322
Laskowska 2015 [24]	sFlt-1	After clinical recognition	2nd-3rd trimester	64 / 65	26.0 (71.4)	2.67 (7.62)	ng/mL	0.46	0.177
Fabjan-Vodušek 2019 [27]	sFlt-1	After clinical recognition	>=24 wk	32 / 12	18,638 (11,770)	8,913 (3,143)	µg/L	0.93	0.348
Schoots 2021 [15]	sFlt-1	After clinical recognition	24-36 wk	11 / 14	14,424 (8,941)	4,655 (5,515)	pg/mL	1.31	0.435
Lorenz-Meyer 2022 [29]	sFlt-1	After clinical recognition	3rd trimester	25 / 40	9,473 (4,611)	6,394 (4,719)	pg/mL	0.65	0.258
Dymara-Konopka 2023 [13]	sFlt-1	After clinical recognition	close to delivery	22 / 14	129,263 (123,234)	51,193 (49,647)	pg/mL	0.75	0.346
Kluivers 2023 [30]	sFlt-1	After clinical recognition	<34+0 wk	23 / 9	8,456 (5,966)	3,356 (3,918)	pg/mL	0.90	0.401
Rajiv 2024 [31]	sFlt-1	After clinical recognition	3rd trimester	82 / 39	11,827 (7,107)	6,849 (5,757)	pg/mL	0.74	0.199
Jungelson 2025 [33]	sFlt-1	After clinical recognition	28+0-33+6 wk	25 / 42	11,037 (5,089)	2,890 (2,089)	pg/mL	2.30	0.321
Youssef 2026 [35]	sFlt-1	After clinical recognition	>=28+0 wk	80 / 300	4.03 (3.34)	1.29 (1.06)	MoM	1.52	0.137
Shibata 2005 [16]	PlGF	After clinical recognition	At diagnosis	5 / 21	113 (111)	149 (101)	pg/mL	−0.34	0.484
Alahakoon 2014 [23]	PlGF	After clinical recognition	24-41 wk	16 / 24	28.7 (31.7)	25.7 (26.0)	pg/mL	0.10	0.317
Herraiz 2014 [14]	PlGF	After clinical recognition	24+0-33+6 wk	22 / 19	31.5 (22.5)	48.8 (33.4)	pg/mL	−0.60	0.315
Fabjan-Vodušek 2019 [27]	PlGF	After clinical recognition	>=24 wk	32 / 12	68.4 (44.6)	82.1 (89.1)	µg/L	−0.22	0.333
Schoots 2021 [15]	PlGF	After clinical recognition	24-36 wk	11 / 14	11.2 (8.23)	102 (92.7)	pg/mL	−1.25	0.431
Lorenz-Meyer 2022 [29]	PlGF	After clinical recognition	3rd trimester	25 / 40	42.3 (30.7)	52.7 (37.7)	pg/mL	−0.29	0.253
Dymara-Konopka 2023 [13]	PlGF	After clinical recognition	close to delivery	22 / 14	72.0 (62.0)	216 (261)	pg/mL	−0.84	0.349
Kluivers 2023 [30]	PlGF	After clinical recognition	<34+0 wk	23 / 9	23.3 (22.9)	314 (510)	pg/mL	−1.07	0.407
Rajiv 2024 [31]	PlGF	After clinical recognition	3rd trimester	82 / 39	76.6 (83.2)	180 (179)	pg/mL	−0.85	0.201
Jungelson 2025 [33]	PlGF	After clinical recognition	28+0-33+6 wk	25 / 42	114 (45.6)	500 (552)	pg/mL	−0.87	0.261
Shinohara 2026 [34]	PlGF	After clinical recognition	22+0-33+6 wk	65 / 267	75.5 (98.2)	399 (396)	pg/mL	−0.90	0.142
Youssef 2026 [35]	PlGF	After clinical recognition	>=28+0 wk	80 / 300	0.49 (0.42)	0.65 (0.64)	MoM	−0.27	0.126
Diab 2008 [19]	sFlt-1/PlGF ratio	Before clinical recognition	Mean 23 wk	8 / 9	29.2 (22.2)	11.9 (9.06)	ratio	0.99	0.496
Savvidou 2008 [20]	sFlt-1/PlGF ratio	Before clinical recognition	23-25 wk	5 / 15	24.4 (39.7)	5.13 (6.30)	ratio	0.94	0.519
Kienast 2016 [25]	sFlt-1/PlGF ratio	Before clinical recognition	18-25 wk	14 / 26	10.2 (6.50)	8.70 (4.80)	ratio	0.27	0.326
Honigberg 2018 [26]	sFlt-1/PlGF ratio	Before clinical recognition	Mean 26 wk	47 / 238	62.6 (87.0)	19.3 (15.7)	ratio	1.14	0.166
Wathen 2006 [18]	sFlt-1	Before clinical recognition	16-20 wk	8 / 10	585 (673)	366 (194)	ng/L	0.45	0.459
Diab 2008 [19]	sFlt-1	Before clinical recognition	Mean 23 wk	8 / 9	2,562 (1,611)	1,196 (658)	pg/mL	1.08	0.502
Kienast 2016 [25]	sFlt-1	Before clinical recognition	18-25 wk	14 / 26	2,158 (1,308)	1,981 (706)	pg/mL	0.18	0.326
Honigberg 2018 [26]	sFlt-1	Before clinical recognition	Mean 26 wk	47 / 238	8,933 (7,036)	7,083 (4,773)	pg/mL	0.35	0.160
Diab 2008 [19]	PlGF	Before clinical recognition	Mean 23 wk	8 / 9	101 (23.7)	118 (25.9)	pg/mL	−0.64	0.476
Savvidou 2008 [20]	PlGF	Before clinical recognition	23-25 wk	5 / 15	65.2 (29.5)	224 (198)	pg/mL	−0.87	0.515
Cowans 2010 [21]	PlGF	Before clinical recognition	11+0-13+6 wk	7 / 8	0.79 (0.16)	0.51 (0.22)	MoM	1.35	0.554
Ghosh 2013 [22]	PlGF	Before clinical recognition	20-22 wk	11 / 21	135 (25.4)	140 (19.1)	pg/mL	−0.24	0.364
Kienast 2016 [25]	PlGF	Before clinical recognition	18-25 wk	14 / 26	236 (87.0)	264 (113)	pg/mL	−0.26	0.326
Honigberg 2018 [26]	PlGF	Before clinical recognition	Mean 26 wk	47 / 238	204 (179)	407 (263)	pg/mL	−0.81	0.163
Zhang 2019 [28]	PlGF	Before clinical recognition	11+0-13+6 wk	15 / 84	20.4 (16.4)	38.1 (21.1)	pg/mL	−0.86	0.285
Zhang 2024 [32]	PlGF	Before clinical recognition	2nd trimester	40 / 186	80.6 (40.8)	114 (86.8)	pg/mL	−0.41	0.175

PE, preeclampsia; FGR, fetal growth restriction; SGA, small for gestational age; SD, standard deviation; SE, standard error.

One row is presented per study, marker, and sampling context. Means and standard deviations are shown as entered into the analysis. Positive values of Hedges' g indicate higher marker levels in PE-associated FGR/SGA.

Supplementary Table S3. Exploratory univariable meta-regression on study-level moderators, by clinical sampling context

Sampling context	Marker	Moderator	B (95% CI)	p
Before clinical recognition	sFlt-1	Maternal age (per year)	not estimable	—
Before clinical recognition	sFlt-1	Gestational age at sampling (per week)	−0.00 (−0.11 to 0.10)	0.928
Before clinical recognition	sFlt-1	FGR/SGA definition (birthweight-defined SGA vs antenatally defined FGR)	−0.75 (−1.82 to 0.32)	0.172
Before clinical recognition	sFlt-1	Assay class (research ELISA vs automated immunoassay)	0.41 (−0.34 to 1.16)	0.284
Before clinical recognition	sFlt-1	Clinical status at the time of sampling	0.75 (−0.32 to 1.82)	0.172
Before clinical recognition	PlGF	Maternal age (per year)	0.04 (−0.12 to 0.20)	0.597
Before clinical recognition	PlGF	Gestational age at sampling (per week)	−0.05 (−0.12 to 0.03)	0.215
Before clinical recognition	PlGF	FGR/SGA definition (birthweight-defined SGA vs antenatally defined FGR)	−0.05 (−0.92 to 0.83)	0.919
Before clinical recognition	PlGF	Assay class (research ELISA vs automated immunoassay)	−0.14 (−0.96 to 0.68)	0.736
Before clinical recognition	PlGF	Clinical status at the time of sampling	−0.36 (−1.31 to 0.60)	0.462
Before clinical recognition	sFlt-1/PlGF ratio	Maternal age (per year)	not estimable	—
Before clinical recognition	sFlt-1/PlGF ratio	Gestational age at sampling (per week)	0.17 (0.02 to 0.32)	0.028
Before clinical recognition	sFlt-1/PlGF ratio	FGR/SGA definition (birthweight-defined SGA vs antenatally defined FGR)	−0.18 (−1.60 to 1.24)	0.804
Before clinical recognition	sFlt-1/PlGF ratio	Assay class (research ELISA vs automated immunoassay)	0.19 (−0.97 to 1.35)	0.747
Before clinical recognition	sFlt-1/PlGF ratio	Clinical status at the time of sampling	0.19 (−0.97 to 1.35)	0.747
After clinical recognition	sFlt-1	Maternal age (per year)	−0.07 (−0.20 to 0.07)	0.335
After clinical recognition	sFlt-1	Gestational age at sampling (per week)	0.04 (−0.10 to 0.17)	0.613
After clinical recognition	sFlt-1	FGR/SGA definition (birthweight-defined SGA vs antenatally defined FGR)	0.95 (0.25 to 1.66)	0.008
After clinical recognition	sFlt-1	Assay class (research ELISA vs automated immunoassay)	−0.05 (−0.73 to 0.64)	0.895
After clinical recognition	sFlt-1	Clinical status at the time of sampling	not estimable	—
After clinical recognition	PlGF	Maternal age (per year)	−0.04 (−0.13 to 0.06)	0.437
After clinical recognition	PlGF	Gestational age at sampling (per week)	0.08 (−0.02 to 0.18)	0.116
After clinical recognition	PlGF	FGR/SGA definition (birthweight-defined SGA vs antenatally defined FGR)	−0.26 (−0.83 to 0.32)	0.378
After clinical recognition	PlGF	Assay class (research ELISA vs automated immunoassay)	0.47 (0.13 to 0.80)	0.006
After clinical recognition	PlGF	Clinical status at the time of sampling	not estimable	—
After clinical recognition	sFlt-1/PlGF ratio	Maternal age (per year)	0.05 (−0.51 to 0.60)	0.866
After clinical recognition	sFlt-1/PlGF ratio	Gestational age at sampling (per week)	−0.09 (−0.42 to 0.25)	0.613
After clinical recognition	sFlt-1/PlGF ratio	FGR/SGA definition (birthweight-defined SGA vs antenatally defined FGR)	0.40 (−0.93 to 1.73)	0.554
After clinical recognition	sFlt-1/PlGF ratio	Assay class (research ELISA vs automated immunoassay)	−0.68 (−1.78 to 0.42)	0.225
After clinical recognition	sFlt-1/PlGF ratio	Clinical status at the time of sampling	not estimable	—

Each row is a separate univariable random-effects meta-regression fitted within one sampling context. B is the change in Hedges' g per unit of the moderator; for the two binary moderators the reference categories are antenatally defined FGR and automated immunoassay. Maternal age was not estimable for before clinical recognition sFlt-1 and the before clinical recognition sFlt-1/PlGF ratio because only two studies had available values. Clinical status at sampling could not be evaluated as a moderator in the analyses after clinical recognition because the coded moderator showed no variation there. Of the 30 marker–moderator combinations, 25 were estimable. No adjustment for multiplicity was applied and all findings are exploratory. CI, confidence interval.

Supplementary Table S4. Newcastle–Ottawa Scale item-level ratings for the 23 included studies, with the scale version applied to each study and the basis for its selection.

Study	NOS version applied	S1	S2	S3	S4	C1	C2	O/E1	O/E2	O/E3	Sel	Comp	Out/ Exp	Total
Shibata 2005 [16]	Case–control	★	–	★	★	★	★	★	★	–	3	2	2	7
Savvidou 2006 [17]	Cohort	–	★	★	★	★	–	★	★	★	3	1	3	7
Wathen 2006 [18]	Case–control	★	★	★	★	★	–	★	★	★	4	1	3	8
Diab 2008 [19]	Cohort	–	★	★	★	★	–	★	★	★	3	1	3	7
Savvidou 2008 [20]	Cohort	–	★	★	★	★	–	★	★	★	3	1	3	7
Cowans 2010 [21]	Case–control	★	–	★	★	★	–	★	★	–	3	1	2	6
Ghosh 2013 [22]	Cohort	★	★	★	★	★	–	★	★	★	4	1	3	8
Alahakoon 2014 [23]	Case–control	★	–	★	★	–	★	★	★	–	3	1	2	6
Herraiz 2014 [14]	Case–control	★	★	★	★	–	–	★	★	★	4	0	3	7
Laskowska 2015 [24]	Case–control	★	–	★	★	–	–	★	★	–	3	0	2	5
Kienast 2016 [25]	Cohort	★	★	★	★	★	–	★	★	★	4	1	3	8
Honigberg 2018 [26]	Cohort	★	★	★	★	★	–	★	★	★	4	1	3	8
Fabjan-Vodušek 2019 [27]	Cohort	–	★	★	–	–	–	★	★	★	2	0	3	5
Zhang 2019 [28]	Cohort	★	★	★	★	★	★	★	★	★	4	2	3	9
Schoots 2021 [15]	Case–control	★	–	★	–	–	–	★	★	–	2	0	2	4
Lorenz-Meyer 2022 [29]	Cohort	–	★	★	–	–	–	–	★	★	2	0	2	4
Dymara-Konopka 2023 [13]	Case–control	★	–	★	★	–	–	★	★	–	3	0	2	5
Kluivers 2023 [30]	Cohort	–	★	★	–	–	–	★	★	★	2	0	3	5
Rajiv 2024 [31]	Case–control	★	★	★	★	–	–	–	★	–	4	0	1	5
Zhang 2024 [32]	Case–control	★	★	★	★	★	–	★	★	–	4	1	2	7
Jungelson 2025 [33]	Cohort	–	★	★	–	–	–	★	★	★	2	0	3	5
Shinohara 2026 [34]	Cohort	–	★	★	–	–	–	★	★	★	2	0	3	5
Youssef 2026 [35]	Cohort	★	★	★	–	★	–	★	★	★	3	1	3	7

★ = star awarded; – = not awarded. The version appropriate to each study's participant selection and sampling structure was applied rather than the design label used by its authors: the cohort version where participants were assembled as a single cohort and classified by phenotype thereafter (13 studies), and the case–control version where the comparison groups were assembled on the basis of disease status and biomarker values were then ascertained (10 studies).

Savvidou 2006 [17] was assessed with the cohort version: three groups were examined at 23–25 weeks of gestation, defined by uterine artery Doppler waveforms and fetal growth status, and preeclampsia was ascertained subsequently. Schoots 2021 [15] was assessed with the case–control version: the comparison groups were defined by the presence of preeclampsia among pregnancies with fetal growth restriction, and biomarker values were then ascertained.

Cohort version — Selection: S1, representativeness of the exposed cohort; S2, selection of the non-exposed cohort; S3, ascertainment of exposure (marker measurement); S4, outcome not present at the start of the study. Outcome: O1, assessment of outcome; O2, adequacy of follow-up duration; O3, completeness of follow-up.

Case–control version — Selection: S1, adequacy of case definition; S2, representativeness of the cases; S3, selection of controls; S4, definition of controls. Exposure: E1, ascertainment of exposure; E2, same method of ascertainment for cases and controls; E3, non-response rate.

Comparability (both versions), specified before assessment: C1, control for gestational age at blood sampling by matching, statistical adjustment, or a protocol-fixed common sampling window, for which matching on gestational age at delivery was not accepted; C2, control for maternal age, body mass index, parity or plurality. A C1 star awarded on a protocol-fixed common window indicates that sampling was constrained by design; it does not establish that the two comparison groups were sampled at the same gestational age, which could not be verified in studies that reported only a common window.

What controlled gestational age at sampling in each study, and whether the study matched, adjusted or applied a protocol-fixed common window, is reported for every study in Supplementary Table S1.

Sel, Comp and Out/Exp are domain subtotals (maxima 4, 2 and 3); Total is out of 9. Totals ranged from 4 to 9 (median 7). In the comparability domain 2 studies received both stars, 11 received one and 10 received neither.

ACOG, American College of Obstetricians and Gynecologists; FGR, fetal growth restriction; FIGO, International Federation of Gynecology and Obstetrics; ISSHP, International Society for the Study of Hypertension in Pregnancy; MoM, multiples of the median; NHBPEP, National High Blood Pressure Education Program; PE, preeclampsia; PlGF, placental growth factor; sFlt-1, soluble fms-like tyrosine kinase-1; SGA, small for gestational age.

Supplementary Table S5. Pooled estimates stratified by growth-outcome definition, by clinical sampling context.

Sampling context	Marker	Growth-outcome definition	Studies (k)	Hedges' g (95% CI)	p	I^2 (%)	Between-definition difference χ^2_1	p
Before clinical recognition	sFlt-1/PlGF ratio	Antenatally defined FGR	1*	0.99 (−0.03 to 2.02)	0.057	—	0.09	0.765
	sFlt-1/PlGF ratio	Birthweight-defined SGA	3	0.81 (0.23 to 1.40)	0.007	63.3		
	sFlt-1	Antenatally defined FGR	1*	1.08 (0.04 to 2.12)	0.041	—	1.87	0.171
	sFlt-1	Birthweight-defined SGA	3	0.33 (0.06 to 0.60)	0.016	0.0		
	PlGF	Antenatally defined FGR	2	−0.38 (−0.97 to 0.20)	0.201	0.0	0.01	0.915
	PlGF	Birthweight-defined SGA	6	−0.42 (−0.88 to 0.03)	0.069	67.5		
After clinical recognition	sFlt-1/PlGF ratio	Antenatally defined FGR	7	0.95 (0.33 to 1.58)	0.003	78.5	0.33	0.563
	sFlt-1/PlGF ratio	Birthweight-defined SGA	2	1.35 (0.15 to 2.56)	0.028	83.0		
	sFlt-1	Antenatally defined FGR	10	0.87 (0.60 to 1.15)	<0.001	70.7	3.61	0.058
	sFlt-1	Birthweight-defined SGA	3	1.83 (0.88 to 2.78)	<0.001	74.6		
	PlGF	Antenatally defined FGR	9	−0.55 (−0.81 to −0.30)	<0.001	64.4	1.30	0.254
	PlGF	Birthweight-defined SGA	3	−0.83 (−1.23 to −0.43)	<0.001	0.0		

CI, confidence interval; FGR, fetal growth restriction; PlGF, placental growth factor; sFlt-1, soluble fms-like tyrosine kinase-1; SGA, small for gestational age.

Estimates are standardised mean differences (Hedges' g) for PE-associated minus isolated FGR/SGA, pooled with random-effects models (restricted maximum likelihood). The growth-outcome definition applied by each study is reported in Supplementary Table S1; 13 studies used antenatally defined FGR and 10 used birthweight-defined SGA.

*Only one study contributed to this stratum, so the entry is that study's own estimate rather than a pooled estimate and no heterogeneity statistic is available.

The between-definition difference is a test for subgroup differences between the two strata, with the between-study variance estimated separately within each stratum (1 degree of freedom).

These stratified estimates are presented to assess the consistency of findings across growth-outcome definitions. Between-subgroup comparisons were exploratory and should be interpreted cautiously because of the small number of studies in several strata; the corresponding moderator analysis is reported in Supplementary Table S3.

Supplementary Table S6. Results of the sensitivity analyses for the six pooled comparisons.

Analysis	Sensitivity analysis	Studies (k)	Hedges' g (95% CI) or interval	I² (%)	Note
Before clinical recognition, sFlt-1/PlGF ratio	Main analysis	4	0.86 (0.38 to 1.33)	45.0	
	Hartung–Knapp inference	4	0.86 (0.19 to 1.52)	—	p = 0.026
	95% prediction interval	4	−0.89 to 2.60	—	Interval for the effect in a new study
	Omitting each study in turn	4	0.60 to 1.11	—	Range of pooled estimates; significant throughout
	Excluding studies with fewer than 10 PE-associated cases	2	0.75 (−0.09 to 1.60)	81.6	
	Excluding case–control designs	4	0.86 (0.38 to 1.33)	45.0	
	Excluding studies scoring below the median of 7 of 9 Newcastle–Ottawa stars	4	0.86 (0.38 to 1.33)	45.0	
	Restricted to directly reported means and standard deviations	2	0.52 (−0.15 to 1.20)	26.9	
	Restricted to studies reporting group-specific sampling gestational ages	1	0.27 (−0.38 to 0.92)	—	Single study; not a pooled estimate
	Restricted to a between-group difference in sampling gestational age of ≤ 1.5 weeks	1	0.27 (−0.38 to 0.92)	—	Single study; not a pooled estimate
	Restricted to documented between-group comparability of sampling gestational age or gestational-age-standardised values	1	0.27 (−0.38 to 0.92)	—	Single study; not a pooled estimate
Before clinical recognition, sFlt-1	Main analysis	4	0.38 (0.12 to 0.64)	0	
	Hartung–Knapp inference	4	0.38 (0.02 to 0.74)	—	p = 0.044
	95% prediction interval	4	−0.20 to 0.96	—	Interval for the effect in a new study
	Omitting each study in turn	4	0.33 to 0.44	—	Range of pooled estimates; not significant when Honigberg 2018 [26] was omitted
	Excluding studies with fewer than 10 PE-associated cases	2	0.32 (0.04 to 0.60)	0.0	
	Excluding case–control designs	3	0.37 (0.10 to 0.65)	6.3	
	Excluding studies scoring below the median of 7 of 9 Newcastle–Ottawa stars	4	0.38 (0.12 to 0.64)	0.0	
	Restricted to directly reported means and standard deviations	2	0.54 (−0.32 to 1.40)	51.7	
	Restricted to studies reporting group-specific sampling gestational ages	1	0.18 (−0.47 to 0.83)	—	Single study; not a pooled estimate
	Restricted to a between-group difference in sampling gestational age of ≤ 1.5 weeks	1	0.18 (−0.47 to 0.83)	—	Single study; not a pooled estimate
	Restricted to documented between-group comparability of sampling gestational age or gestational-age-standardised values	1	0.18 (−0.47 to 0.83)	—	Single study; not a pooled estimate
Before clinical recognition, PlGF	Main analysis	8	−0.46 (−0.77 to −0.16)	56.3	
	Hartung–Knapp inference	8	−0.46 (−0.91 to −0.02)	—	p = 0.044
	95% prediction interval	8	−1.27 to 0.34	—	Interval for the effect in a new study
	Omitting each study in turn	8	−0.59 to −0.38	—	Range of pooled estimates; significant throughout
	Excluding studies with fewer than 10 PE-associated cases	5	−0.56 (−0.81 to −0.32)	23.1	
	Excluding case–control designs	6	−0.67 (−0.91 to −0.44)	0.0	
	Excluding studies scoring below the median of 7 of 9 Newcastle–Ottawa stars	7	−0.59 (−0.81 to −0.36)	0.0	
	Restricted to directly reported means and standard deviations	4	−0.43 (−0.71 to −0.15)	0.0	
	Restricted to studies reporting group-specific sampling gestational ages	1	−0.26 (−0.91 to 0.39)	—	Single study; not a pooled estimate

	Restricted to a between-group difference in sampling gestational age of ≤ 1.5 weeks	1	−0.26 (−0.91 to 0.39)	—	Single study; not a pooled estimate
	Restricted to documented between-group comparability of sampling gestational age or gestational-age-standardised values	3	0.01 (−1.17 to 1.19)	81.4	
After clinical recognition, sFlt-1/PlGF ratio	Main analysis	9	1.04 (0.51 to 1.57)	79.3	
	Hartung–Knapp inference	9	1.04 (0.36 to 1.71)	—	$p = 0.008$
	95% prediction interval	9	−0.81 to 2.88	—	Interval for the effect in a new study
	Omitting each study in turn	9	0.86 to 1.16	—	Range of pooled estimates; significant throughout
	Excluding studies with fewer than 10 PE-associated cases	9	1.04 (0.51 to 1.57)	79.3	
	Excluding case–control designs	5	0.98 (0.47 to 1.48)	73.6	
	Excluding studies scoring below the median of 7 of 9 Newcastle–Ottawa stars	2	1.12 (0.88 to 1.36)	0.0	
	Restricted to directly reported means and standard deviations	1	0.58 (−0.10 to 1.26)	—	Single study; not a pooled estimate
	Restricted to studies reporting group-specific sampling gestational ages	7	1.14 (0.43 to 1.85)	83.0	
	Restricted to a between-group difference in sampling gestational age of ≤ 1.5 weeks	4	1.16 (0.54 to 1.78)	75.2	
	Restricted to documented between-group comparability of sampling gestational age or gestational-age-standardised values	8	1.12 (0.53 to 1.71)	80.4	
After clinical recognition, sFlt-1	Main analysis	13	1.05 (0.73 to 1.38)	76.8	
	Hartung–Knapp inference	13	1.05 (0.69 to 1.42)	—	$p < 0.001$
	95% prediction interval	13	−0.08 to 2.19	—	Interval for the effect in a new study
	Omitting each study in turn	13	0.93 to 1.12	—	Range of pooled estimates; significant throughout
	Excluding studies with fewer than 10 PE-associated cases	11	0.98 (0.65 to 1.31)	78.6	
	Excluding case–control designs	6	1.27 (0.76 to 1.79)	74.7	
	Excluding studies scoring below the median of 7 of 9 Newcastle–Ottawa stars	4	1.42 (0.90 to 1.93)	49.3	
	Restricted to directly reported means and standard deviations	2	0.52 (0.21 to 0.83)	0.0	
	Restricted to studies reporting group-specific sampling gestational ages	7	1.03 (0.55 to 1.50)	72.0	
	Restricted to a between-group difference in sampling gestational age of ≤ 1.5 weeks	4	1.17 (0.42 to 1.93)	82.6	
	Restricted to documented between-group comparability of sampling gestational age or gestational-age-standardised values	8	1.11 (0.69 to 1.53)	76.1	
After clinical recognition, PlGF	Main analysis	12	−0.60 (−0.82 to −0.38)	56.8	
	Hartung–Knapp inference	12	−0.60 (−0.84 to −0.36)	—	$p < 0.001$
	95% prediction interval	12	−1.25 to 0.05	—	Interval for the effect in a new study
	Omitting each study in turn	12	−0.66 to −0.55	—	Range of pooled estimates; significant throughout
	Excluding studies with fewer than 10 PE-associated cases	11	−0.61 (−0.84 to −0.38)	60.4	
	Excluding case–control designs	6	−0.58 (−0.88 to −0.28)	69.0	
	Excluding studies scoring below the median of 7 of 9 Newcastle–Ottawa stars	3	−0.31 (−0.54 to −0.09)	0.0	
	Restricted to directly reported means and standard deviations	2	−0.89 (−1.15 to −0.63)	0.0	
	Restricted to studies reporting group-specific sampling gestational ages	7	−0.63 (−0.97 to −0.30)	45.8	

	Restricted to a between-group difference in sampling gestational age of ≤ 1.5 weeks	4	−0.65 (−0.99 to −0.32)	18.7	
	Restricted to documented between-group comparability of sampling gestational age or gestational-age-standardised values	8	−0.55 (−0.84 to −0.26)	52.7	

CI, confidence interval; PE, preeclampsia; PlGF, placental growth factor; sFlt-1, soluble fms-like tyrosine kinase-1.

Estimates are standardised mean differences (Hedges' g) for PE-associated minus isolated FGR/SGA, pooled with random-effects models (restricted maximum likelihood) and Wald-type confidence intervals unless stated otherwise. For the main analyses the heterogeneity statistics are those reported in the Results.

Hartung–Knapp inference uses a t distribution with k − 1 degrees of freedom; prediction intervals use a t distribution with k − 2 degrees of freedom and are not estimable for fewer than three studies.

Newcastle–Ottawa totals and the scale version applied to each study are reported in Supplementary Table S4; the sampling gestational ages, their between-group differences and the comparability classification are reported in Supplementary Table S1.

Restrictions that leave a single study are reported for completeness: the entry is that study's own estimate rather than a pooled estimate, and no heterogeneity statistic is available. Restricted estimates based on few studies are imprecise and are reported descriptively.

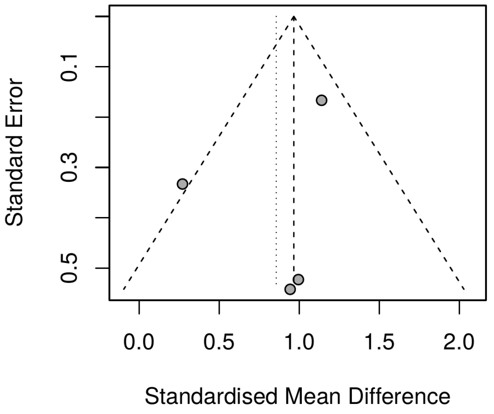

(a) sFlt-1/PlGF ratio, before clinical recognition (Egger p = 0.526)

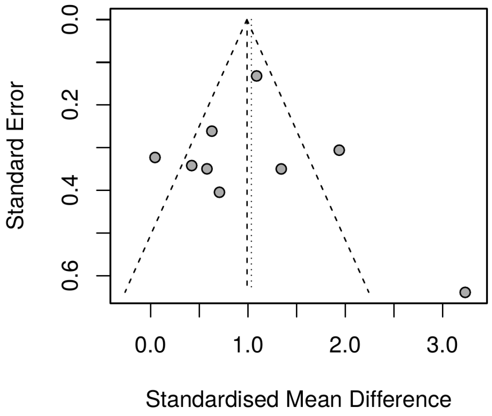

(b) sFlt-1/PlGF ratio, after clinical recognition (Egger p = 0.860)

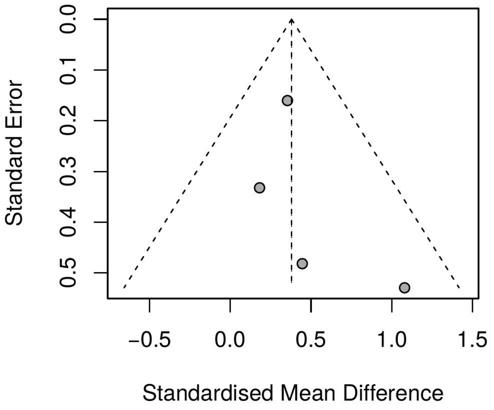

(c) sFlt-1, before clinical recognition (Egger p = 0.485)

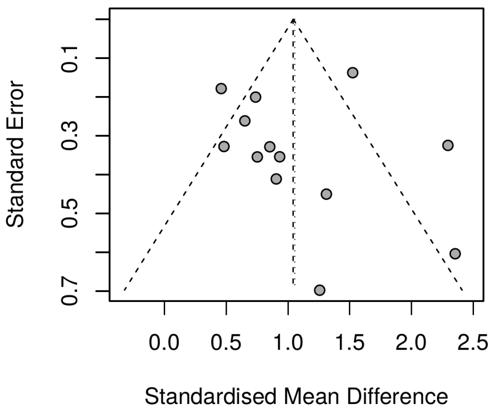

(d) sFlt-1, after clinical recognition (Egger p = 0.912)

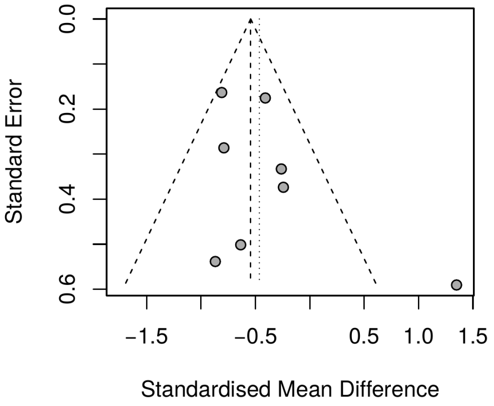

(e) PlGF, before clinical recognition (Egger p = 0.235)

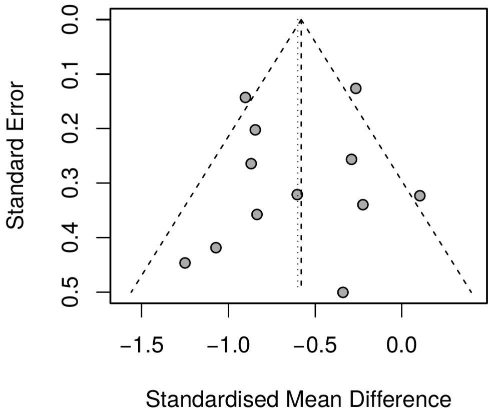

(f) PlGF, after clinical recognition (Egger p = 0.725)

Supplementary Figure S1. Funnel plots for the six pooled analyses

Each point represents one study, plotted as the standardised mean difference (Hedges' g) against its standard error. Vertical lines indicate the pooled estimates and the diagonal dashed lines the pseudo 95% confidence limits.

PlGF, placental growth factor; sFlt-1, soluble fms-like tyrosine kinase-1.
